# Dipolar Excitonic Quantum Wires at Atomically Sharp Lateral Interfaces

**DOI:** 10.1021/acs.nanolett.6c02624

**Published:** 2026-09-15

**Authors:** Elie Vandoolaeghe, Francesco Fortuna, Suman Kumar Chakraborty, Biswajeet Nayak, Takashi Taniguchi, Kenji Watanabe, Prasana K. Sahoo, Thibault Chervy, Puneet A. Murthy

**Affiliations:** † Institute for Quantum Electronics, ETH Zürich, CH-8093 Zürich, Switzerland; ‡ Quantum Materials and Device Research Lab, Materials Research Center, Indian Institute of Technology, Kharagpur 721302, India; § 52747 National Institute for Materials Science, Namiki 1-1, Tsukuba 305-0044, Ibaraki, Japan; ∥ NTT Research, Inc. Physics & Informatics Laboratories, 940 Stewart Dr, Sunnyvale, California 94085, United States

**Keywords:** 1D excitons, lateral heterostructures, quantum confinement, dipolar excitons

## Abstract

One-dimensional (1D) quantum systems are a cornerstone of condensed matter physics. However, their realization in solids has traditionally relied on top-down methods, which are limited by structural disorder and coarse confinement. Here, we demonstrate a fundamentally new route to low-dimensional systems: the emergence of 1D quantum matter at the atomically sharp interface between monolayer semiconductors. Using lateral MoSe_2_–WSe_2_ heterostructures, we identify interfacial excitonic quasiparticles bound to the crystal junction. Photoluminescence spectroscopy resolves these excitons into a ladder of discrete states, establishing 1D confinement at length scales **≲**3 nm. These excitons possess exceptionally large permanent in-plane electric dipole moments exceeding *e* × 2 nm, and exhibit micron-scale anisotropic diffusion confined to the interface. By introducing electrostatic doping, we demonstrate a collapse of the dipole moment and a 20-fold reduction in lifetime. By dynamically tuning wavefunctions within a single atomic monolayer, we open a scalable route toward engineering 1D quantum matter.

## Graphical abstract

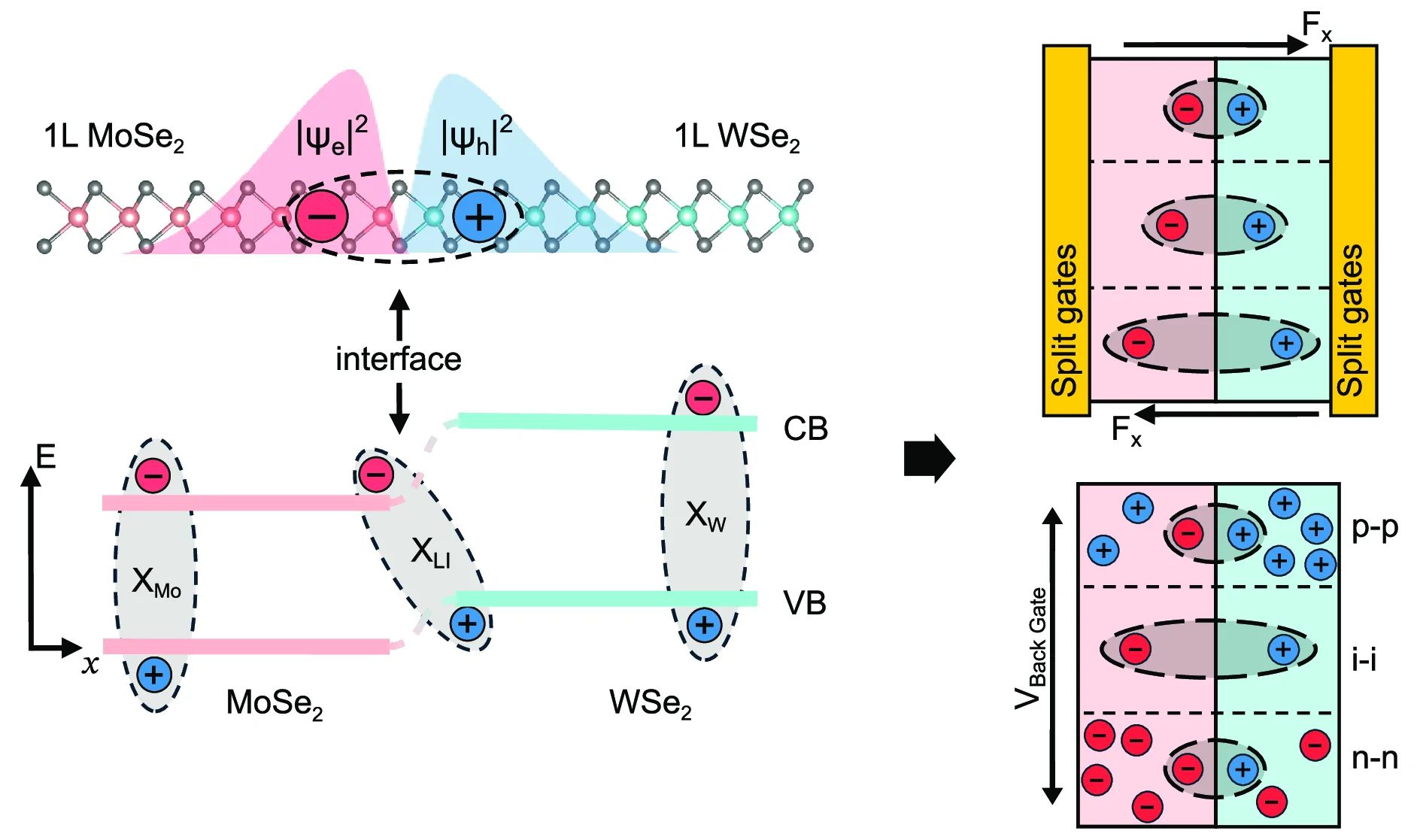

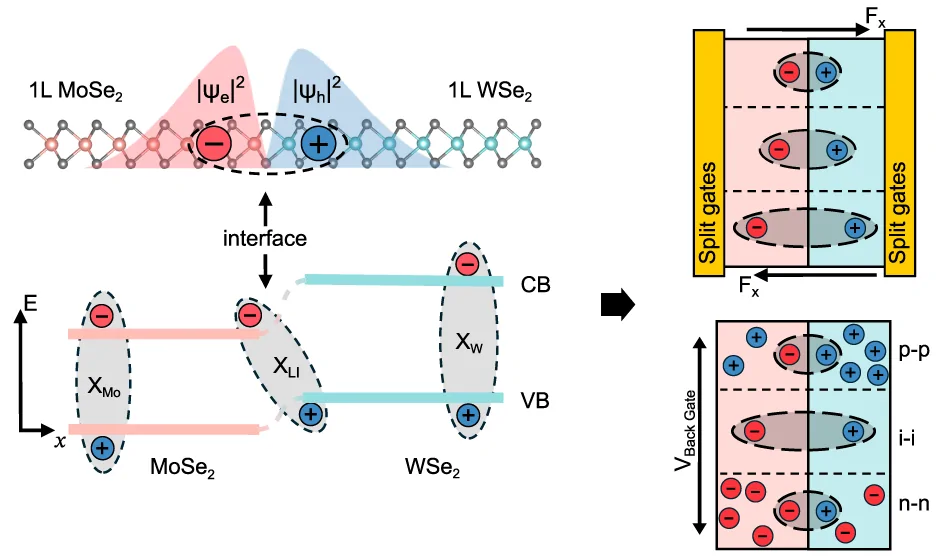

The realization of one-dimensional (1D) quantum systems in solids remains a central objective in condensed-matter physics. In the 1D limit, reduced dimensionality enhances interactions and correlations, giving rise to distinct quantum phases such as Luttinger liquids and Tonks–Girardeau gases.^1,2^ At the same time, the restricted phase space for scattering can enable transport regimes with suppressed phonon and disorder interactions, offering the prospect of high-mobility channels beyond what is achievable in higher dimensions.^3^ In solid-state systems, 1D channels are typically engineered by externally imposing confinement through top-down lithography or electrostatic gating.^4–7^ However, such approaches inherently rely on mesoscale patterning and are therefore limited by extrinsic disorder, fabrication constraints, and confinement lengths that remain significantly larger than the intrinsic size of the quasiparticles.

We demonstrate a fundamentally distinct paradigm: the intrinsic emergence of 1D quantum wires of excitons at the atomically sharp interface between dissimilar transition-metal dichalcogenide (TMD) monolayers, MoSe_2_ and WSe_2_ (Figure 1a). van der Waals (vdW) semiconductors are uniquely suited for this approach, providing boundaries defined with near-atomic precision. Recent advances in chemical vapor deposition (CVD) enable the synthesis of lateral heterostructures (LHS) where two monolayers are seamlessly stitched within a single atomic plane.^8–11^ A micrograph of a typical LHS flake is shown in Figure 1b. These junctions form atomically sharp interfaces only a few lattice constants wide yet extending over tens of microns, realizing clean, crystallographically defined 1D boundaries between 2D electronic phases. The atomic sharpness is confirmed via HAADF-STEM imaging of LHS flakes, shown in Figure 1c.

**Figure 1:**
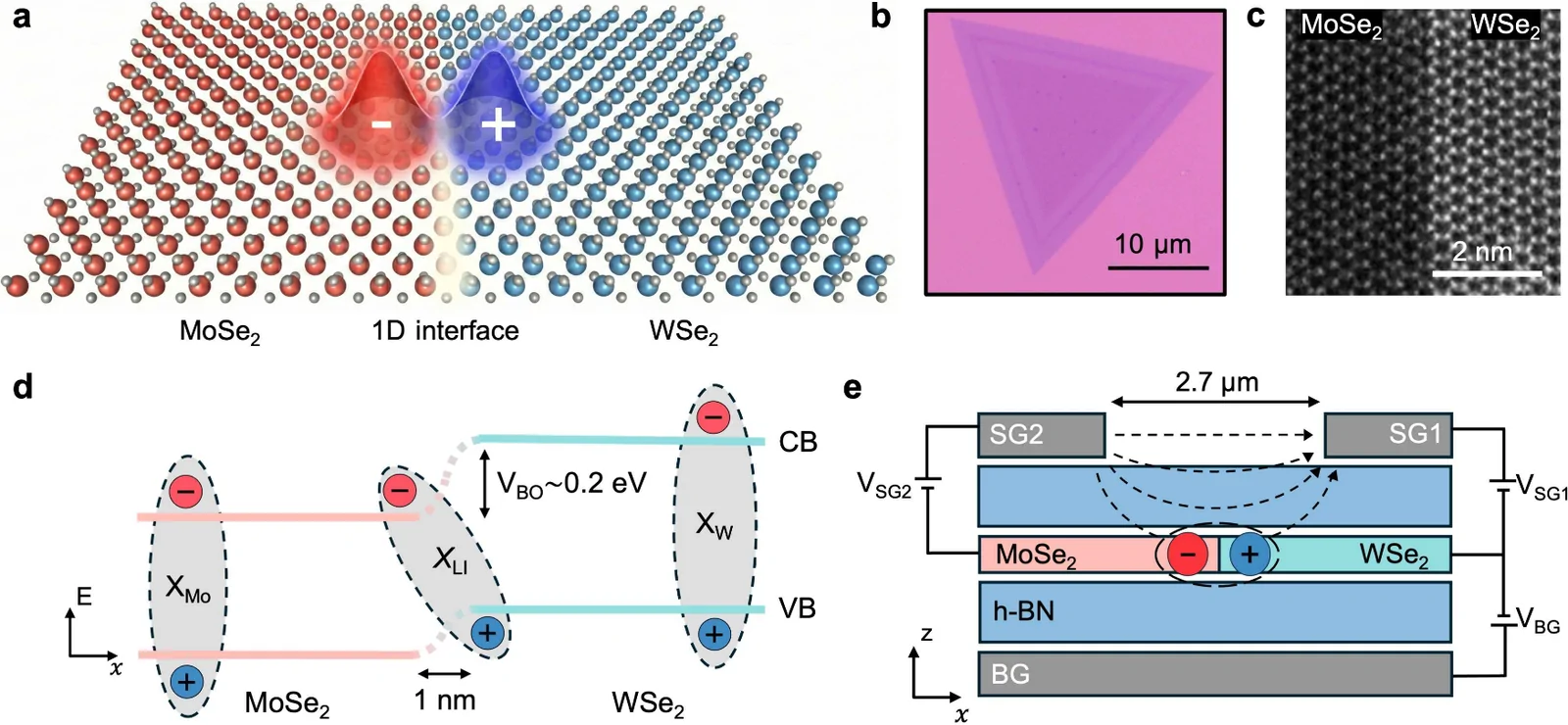
Lateral interfaces and the interfacial dipolar exciton (*X*_LI_). **a**, Illustration of the 1D lateral interface between 2D semiconductors MoSe_2_ and WSe_2_, hosting interfacial excitons. **b**, Micrograph of CVD-grown monolayer lateral heterostructures (LHS) of MoSe_2_ and WSe_2_. **c**, High Angle Annular Dark-Field Scanning Transmission Electron Microscopy (HAADF-STEM) image confirming the atomic sharpness of the interface. **d**, Band edge diagram illustrating the Type-II alignment, confining the electron in MoSe_2_ and the hole in WSe_2_. *X*_Mo_ and *X*_W_ denote the bulk direct excitons. The predicted state *X*_LI_ is the interfacial dipolar exciton, where the separated charges remain Coulomb-bound at the junction. **e**, Schematic of the device architecture: h-BN (top and bottom are 25 nm thick) encapsulated monolayer LHS, featuring bottom (BG) and split top gates (SG1, SG2) for control over carrier density and in-plane electric field.

The elementary optical excitations in these systems are excitons—Coulomb-bound electron-hole pairs. In monolayer TMDs, these quasiparticles possess exceptionally high binding energies and their 2D physics is well understood.^12^ While prior works have demonstrated 1D confinement in various lithographically defined or strain-induced nanostructures,^13,14^ these typically remain in the ‘weak’ confinement regime, where the confinement length scale (*l* ≳ 10 nm) far exceeds the intrinsic exciton Bohr radius (*a*_*B*_ ≈ 1 nm). The introduction of a lateral interface, however, introduces a new energy scale from the Type-II band alignment (Figure 1d).^15^ This band offset partitions electrons and holes into separate regions, directly competing with the Coulomb attraction.

When this band offset is comparable to the Coulomb energy, theory predicts^16–21^ the emergence of a new ground state energetically below the 2D exciton of both parent materials. In this unique charge-transfer state, the electron and hole reside on adjacent sides of the junction while remaining Coulomb-bound. These interfacial excitons (*X*_LI_) feature reduced binding energies but exceptionally large, permanent in-plane dipole moments. Crucially, while the carriers are spatially separated, the exciton’s center-of-mass is strictly bound to the 1D junction. Unlike vertical vdW heterostructures^22–24^ —where the dipole is geometrically “frozen” by the layer spacing—this 2D architecture enables the dipole to exist within the junction plane, providing a uniquely tunable platform for the in-situ engineering of 1D quantum matter.

Despite intense theoretical and experimental interest, the realization of 1D quantum-confined dipolar interfacial excitons has been elusive. While prior works have reported preliminary evidence for charge transfer (CT) excitons^19^ as well as exciton transport in LHS,^25–28^ they have been limited by interface width and disorder, leading to broad and weak spectral features.^27,29^ Consequently, the ability to resolve the discrete quantum structure and actively engineer the radiative dynamics of these states has remained an outstanding challenge.

In this work, we realize the regime of strongly quantum-confined 1D dipolar excitons by bridging atomic-scale structural precision with active electrostatic control. To preserve the near-atomic sharpness of the junctions and reduce effects of ambient dielectric disorder, the LHS monolayer flakes are encapsulated in hexagonal boron nitride and integrated into a dual-gate architecture (Figure 1e). This configuration—featuring a global back gate (BG) and split top gates (SG_1_, SG_2_)—enables the independent regulation of carrier density and in-plane electric fields, a critical capability for probing the exciton’s internal structure. By combining high-resolution spatiotemporal spectroscopy with this multi-gate control, we provide an unambiguous demonstration of both the fundamental 1D nature and the functional tunability of these interfacial states.

We begin by experimentally identifying the interfacial exciton state and establishing its spatial, spectral, and temporal signatures at the 1D junction. In Figure 2a, we present normalized photoluminescence (PL) spectra from the 2D MoSe_2_ region (red) and the interface (blue). While the 2D spectrum exhibits the characteristic signatures of the neutral exciton (*X*_Mo_) and trion $\left(X_{\mathrm{Mo}}^{-}\right)$ , measurements at the interface reveal a dominant new resonance at $E_{X_{\mathrm{LI}}}\approx 1.53\mathrm{eV}$ . This peak is absent in the bulk and lies nearly 100 meV below the MoSe_2_ exciton, placing it well below any known optically active states or defect-bound excitons in the parent monolayers.^30,31^ This significant redshift is consistent with the predicted Type-II band alignment and represents the clear spectral fingerprint of a spatially indirect interfacial state.^19^

**Figure 2:**
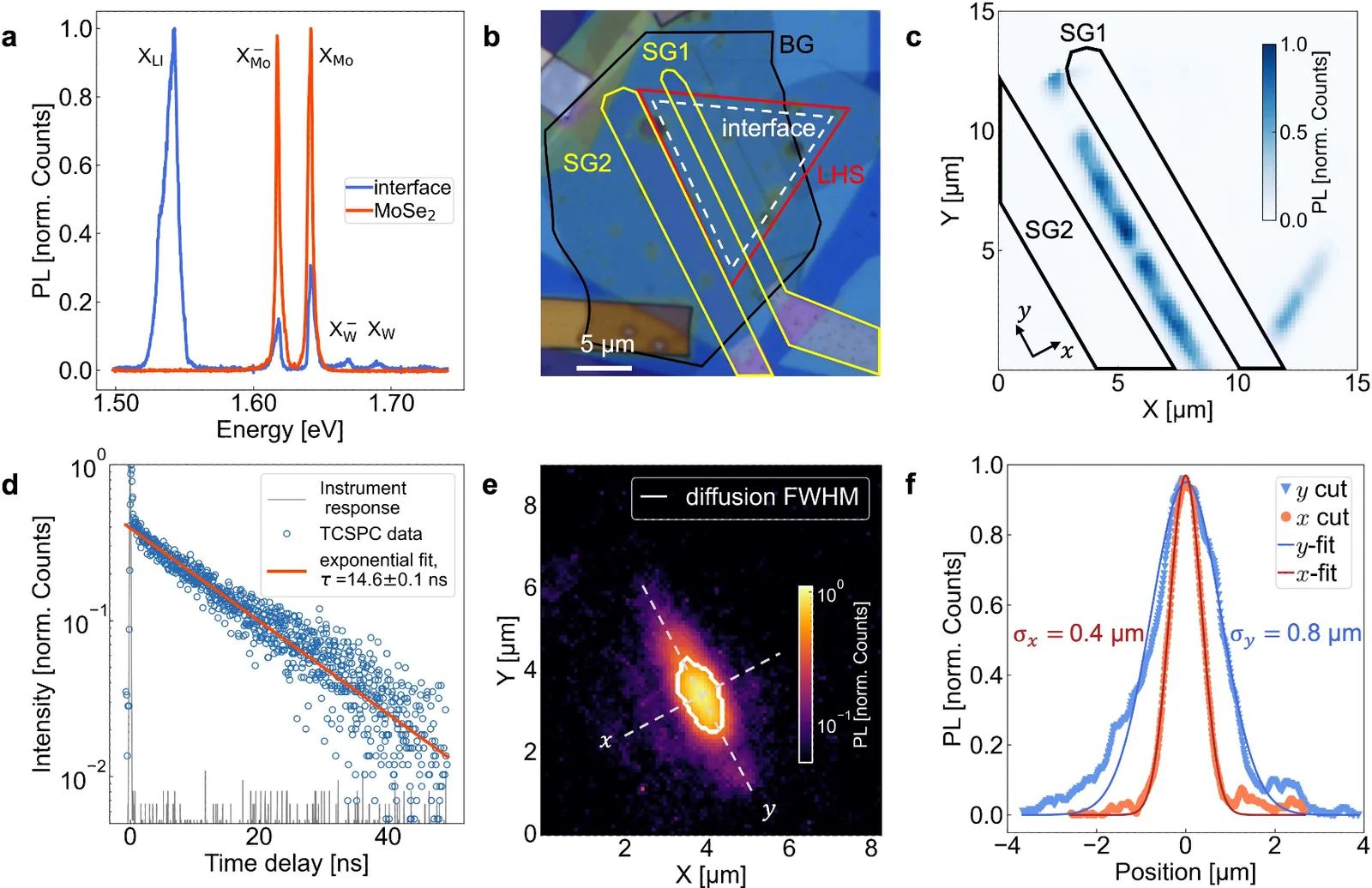
The interfacial exciton in the lateral heterostructure. **a**, Normalized PL spectra at 4 K from the 2D bulk MoSe_2_ (red) and from the MoSe_2_–WSe_2_ interface (blue). A new state, *X*_LI_, emerges at the interface at ≈1.53eV ( ∼100meV below the 2D neutral exciton *X*_Mo_). **b**, Micrograph of the device: The LHS flake, interface, and gate electrodes (BG, SG) are outlined. **c**, Position-resolved PL integrated over *X*_LI_ demonstrates that the state exists exclusively at the 1D interface. **d**, Time-correlated single-photon counting (TCSPC) trace of the *X*_LI_ emission. Fitting the long-time decay yields a lifetime of *τ* = 14.6 ns. **e**, Spatial PL diffusion map of the *X*_LI_ state obtained by wide-field imaging. The white contour is the Full Width at Half Maximum (FWHM) of the emission spot. **f**, Line cuts of the diffusion profile along the short (*x*) and long (*y*) axes. Gaussian fits confirm pronounced anisotropic exciton diffusion, yielding a diffusion length of *L*_*D*_ ≈ 0.7 *μ* m along *y* and an estimated diffusion coefficient of *D*_*y*_ ∼ 0.4 cm^2^s^–1^.

## Spatial Structure

To map the spatial distribution of this emission, we perform position-resolved PL imaging across the heterostructure. For reference, a micrograph of the device is shown in Figure 2b, with outlines of the LHS flake (red) and interface (dashed white). As shown in Figure 2c, the *X*_LI_ emits exclusively from the 1D junction region, extending continuously along its ∼20 *μ* m length. The emission pattern is convolved with the point-spread function of the imaging system, leading to a diffraction limited width. We attribute variations in local intensity to optical absorption by the gold split-gate electrode SG1, and quenching near the graphene contact, located at the top right corner of the flake.^32^ These spatially resolved measurements unambiguously demonstrate that *X*_LI_ is not a local defect but a delocalized 1D state emerging from the crystallographic boundary.

## Lifetime

To probe the temporal dynamics of this state, we employ time-correlated single-photon counting (TCSPC). Fitting the filtered *X*_LI_ emission with a single-exponential decay model yields an interfacial exciton lifetime of *τ* = 14.6 ± 0.1 ns (Figure 2d). This value is three orders of magnitude longer than the lifetimes of intralayer exciton species in the same system (∼1‐100ps) .^33^ This prolonged lifetime is the hallmark of a charge-transfer exciton state: the spatial separation of the electron and hole wavefunctions across the interface reduces their radiative recombination rate, enhancing their lifetime (see the Supporting Information (SI) for details).

## Mobility

We further examine the spatial diffusion of these 1D excitons under fixed continuous-wave (CW) excitation. The mobility of *X*_LI_ serves as a critical metric to confirm their nature as genuine interfacial quasiparticles. Steady-state PL imaging of the *X*_LI_ (Figure 2e) reveals a markedly anisotropic ‘cigar-shaped’ profile, with exciton spreading occurring primarily in the longitudinal (*y*) direction. Line cuts taken along *x* and *y* yield half-widths of (standard deviations, see SI) *σ*_*y*_ = 0.8 ± 0.04 *μ* m and *σ*_*x*_ = 0.4 ± 0.04 *μ*m, respectively. While the transverse width *σ*_*x*_ remains near the diffraction limit of our system (*σ*_PSF_ = 0.35 *μ*m), the longitudinal spread *σ*_*y*_ shows a macroscopic signature of 1D motion.

We determine the longitudinal diffusion length by deconvolving the system’s point-spread function:^34–36^ $L_{D}=\sqrt{\sigma_{y}^{2}-\sigma_{PSF}^{2}}=0.72\pm 0.06$ *μ*m. Using the measured exciton lifetime (*τ* = 15 ns), we estimate an effective 1D diffusion coefficient $D_{y}=L_{D}^{2}/\tau \approx 0.4{\mathrm{cm}}^{2}/s$ . In the low-excitation power regime, where *D*_*y*_ is independent of pump power, we estimate a corresponding exciton mobility *μ*_*X*_ = *eD*_*y*_/*k*_B_ *T* ≈ 1, 200 cm^2^/(V s) at *T* = 4 K.

We emphasize that steady-state imaging provides a time-integrated assessment of diffusion, and further time-resolved measurements would be required to fully map diffusion dynamics. Nonetheless, such measurements indicate a high-quality interfacial 1D channel.^37,38^ In contrast to 2D monolayers, where ubiquitous small-angle scattering limits mobility, 1D kinematics restrict momentum relaxation to discrete backscattering events. This effectively filters out the long-range potential fluctuations common in van der Waals heterostructures,^39,40^ suggesting that the seamless lateral interface provides a uniquely protected environment for excitonic diffusion. Taken together, these signatures provide compelling evidence for long-lived, mobile interfacial excitonic quasiparticles.

We now investigate the properties of the interfacial exciton wavefunction, both in the center-of-mass (COM) and the internal relative degrees of freedom. A closer examination of the *X*_LI_ lineshape in Figure 2a  suggests a fine structure. By reducing the excitation power (≲ 100 nW), thus minimizing power-broadening, we observe that the broad emission resolves into a ladder of narrow, discrete peaks with line widths Γ ≈ 1-3 meV, as shown in Figure 3a. While individual peak energies vary slightly across the sample, the characteristic ladder structure and energy splittings are highly reproducible along the entire junction and across multiple devices (see SI). The interfacial exciton’s center-of-mass is predicted to be confined at the junction, which acts as an effective 1D potential well. This produces a discrete ladder of bound COM states, analogous to a particle in a potential well.

**Figure 3:**
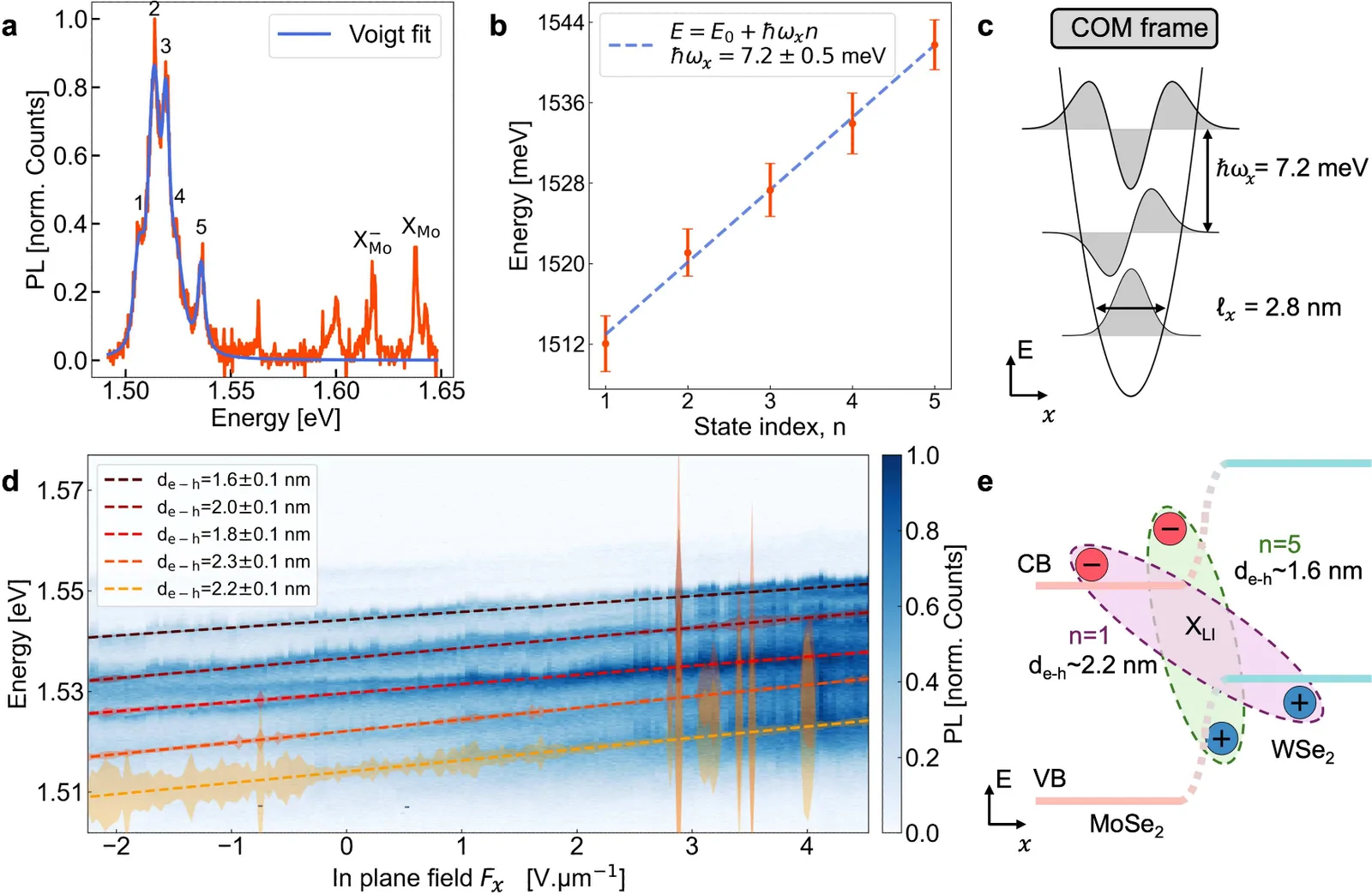
Quantized 1D Motion and Permanent Dipole Moment of the Interfacial Exciton. **a**, Low-power PL spectra showing the *X*_LI_ state resolved into a ladder of narrow, discrete quantum peaks—an unambiguous signature of motional quantization. **b**, Energy of the discrete states (*E*_*n*_) versus state index (*n*). A linear fit (blue dashed line), *E*_*n*_ = *E*_0_ + *ℏω*_*x*_ *n*, yields a harmonic level spacing of *ℏω*_*x*_ = 7.2 ± 0.5 meV, implying a transverse confinement length of *l*_*x*_ ≈ 2.8 nm. **c**, Illustration of the exciton COM wavefunctions confined in a parabolic potential imposed by the band offset. **d**, PL spectra as a function of the in-plane electric field $\left({\mathrm{F⃗}}_{x}\right)$ applied perpendicular to the interface via the split gates. The clear, systematic linear shift (Stark shift) of the peak energies is the definitive signature of a permanent in-plane dipole moment. Linear fits (dashed lines) yield the dipole length $d_{e‐h}^{\left(n\right)}$ . **e**, Schematic illustrating the reduction of extracted dipole lengths with increasing energy, from *d*_e‑h_ ≈ 2.2 nm for the lowest state to ≈1.6nm for higher COM states.

The confinement arises from the Type-II band alignment: as the exciton moves away from the junction, the carriers encounter steep band-edge barriers. The exciton thus experiences an effective confining potential:

$$V_{conf}\left(X\right)=-V_{vb}\left(x_{h}\right)+V_{cb}\left(x_{e}\right)$$

where *V*_vb_ and *V*_cb_ are the valence and conduction band offsets, *x*_*h*_ and *x*_*e*_ are coordinates of hole and electron, and X = (*m*_*e*_ *x*_*e*_ + *m*_*h*_ *x*_*h*_)/(*m*_*e*_ + *m*_*h*_) denotes the exciton’s COM coordinate. Consequently, the potential inherits the full band-offset energy scale (≈0.1−0.2eV) and the atomic-scale width of the interface (independently measured by HAADF-STEM), creating a remarkably deep and tight 1D trap for the bound state. Ultimately, the appearance of this discrete ladder constitutes a definitive spectroscopic signature of quantized center-of-mass (COM) motion,^13,41^ establishing *X*_LI_ as an intrinsically 1D quantum wire state.

In Figure 3b, we plot the center energies of these states extracted from Voigt fits averaged across seven different spatial positions. The energies follow a linear trend, *E*_*n*_ = *E*_0_ + *ℏω*_*x*_ *n*, for the lowest states, yielding a harmonic level spacing of *ℏω*_*x*_ = 7.2 ± 0.5 meV. This corresponds to a nanoscopic transverse confinement length of $l_{x}=\sqrt{\hbar /\left(m_{X}\omega_{x}\right)}=2.8\pm 0.1nm$ (using *m*_X_ = 1.3 *m*_0_) as shown in Figure 3c. Although the underlying band offset step is atomically sharp, the effective COM potential experienced by the exciton is smoothed over the spatial extent of the bound electron–hole pair, yielding an approximately harmonic confinement for the lowest-energy states. When compared to the micron-scale longitudinal diffusion, this yields an extraordinary aspect ratio exceeding 500:1, underscoring the 1D character of the *X*_LI_ state.

Next, we probe the in-plane dipole moment, $\mathrm{p⃗}=e{\mathrm{d⃗}}_{e‐h}$ , of the *X*_LI_ exciton. By applying voltage (*V*_SG1_, *V*_SG2_) on split-gate electrodes (Figure 1e), we apply a controlled in-plane electric field *F*_*x*_ = (*V*_SG1_ – *V*_SG2_)/*D*_SG_ perpendicular to the interface, where *D*_SG_ = 2.7 *μ*m is the split-gate separation. We ensure that these measurements are always performed at neutrality (SI). For a dipole with a fixed orientation, an anti-aligned field produces a blue shift, while an aligned field induces a red shift. The PL spectra as a function of *F*_*x*_ (Figure 3d) reveal a systematic linear Stark shift—the definitive signature of a permanent dipole moment. Linear fits, *E*_*n*_ = *E*_0_ + *e d*_e‑h_(*n*)*F*_*x*_, allow us to precisely extract the permanent dipole length *d*_e‑h_(*n*) for each COM state. These fits yield dipole lengths of *d*_e‑h_ ∼ 2.2 nm for the lowest state (*n* = 1), decreasing systematically to ∼1.6nm for the highest state (*n* = 5), as summarized in Figure 3e. This dipole length is exceptionally large for a bound exciton in TMDs, implying strong dipole–dipole interactions that scale as $U\propto d_{e‐h}^{2}$ .

Critically, since the dipole length is comparable to the transverse confinement scale (*l*_*x*_ ≈ *d*_e‑h_), our observations place the interfacial exciton in the strong-confinement regime. In this limit, the conventional separation of COM and relative motion breaks down; the quasiparticle must be treated as a composite object whose internal wavefunction is “reshaped” by the external potential. The systematic variation of dipole length across the *n*-states is a direct manifestation of this coupled COM-relative dynamics.^42^ This regime stands in sharp contrast to traditional 1D platforms—such as GaAs T-wires or gate-defined TMD wires—where *l*_*x*_ ≫ *a*_*B*_, and the internal structure remains effectively “frozen.” Multiple experimental signatures confirm that these states originate from the intrinsic properties of the interface, rather than from localized defects or disorder. First, the level structure is reproducible across different locations along the interface and different samples, consistently exhibiting 5–6 states separated by ≈7meV . In contrast, disorder-induced or defect-bound quantum dots typically manifest at random spatial positions and energies. Second, we reproducibly observe diffusion of the interfacial excitons over micron-scale distances—an impossible behavior for an ensemble of defects, with nanometer-scale tight binding potentials. Finally, all states exhibit a consistent electric dipole moment, red-shifting simultaneously under an applied in-plane electric field, and the energy shift scaling predictably with the state index. Taken together, these three observations exclude a random distribution of localized states and establish a shared, intrinsic origin.

Having established the fundamental properties of the interfacial exciton, we now examine its response to electrostatic doping and demonstrate the tunability of its dipolar nature and radiative lifetime over an exceptionally wide range.

In Figure 4a, we present the *X*_LI_ photoluminescence (PL) spectra as a function of back-gate voltage (*V*_BG_). At charge neutrality (*V*_BG_ ≈ 0 V), the spectrum exhibits the discrete ladder of COM states. Upon introducing either electron (*V*_BG_ > 0.5 V) or hole (*V*_BG_ < – 2 V) doping, the *X*_LI_ state undergoes a sharp, continuous blue shift of Δ*E* ≈ 70 meV within a narrow voltage window (∼1V) . This shift eventually plateaus at higher voltages, approaching the 2D trion energy of monolayer MoSe_2_, as global charge accumulation begins to screen the interface band offset.

**Figure 4:**
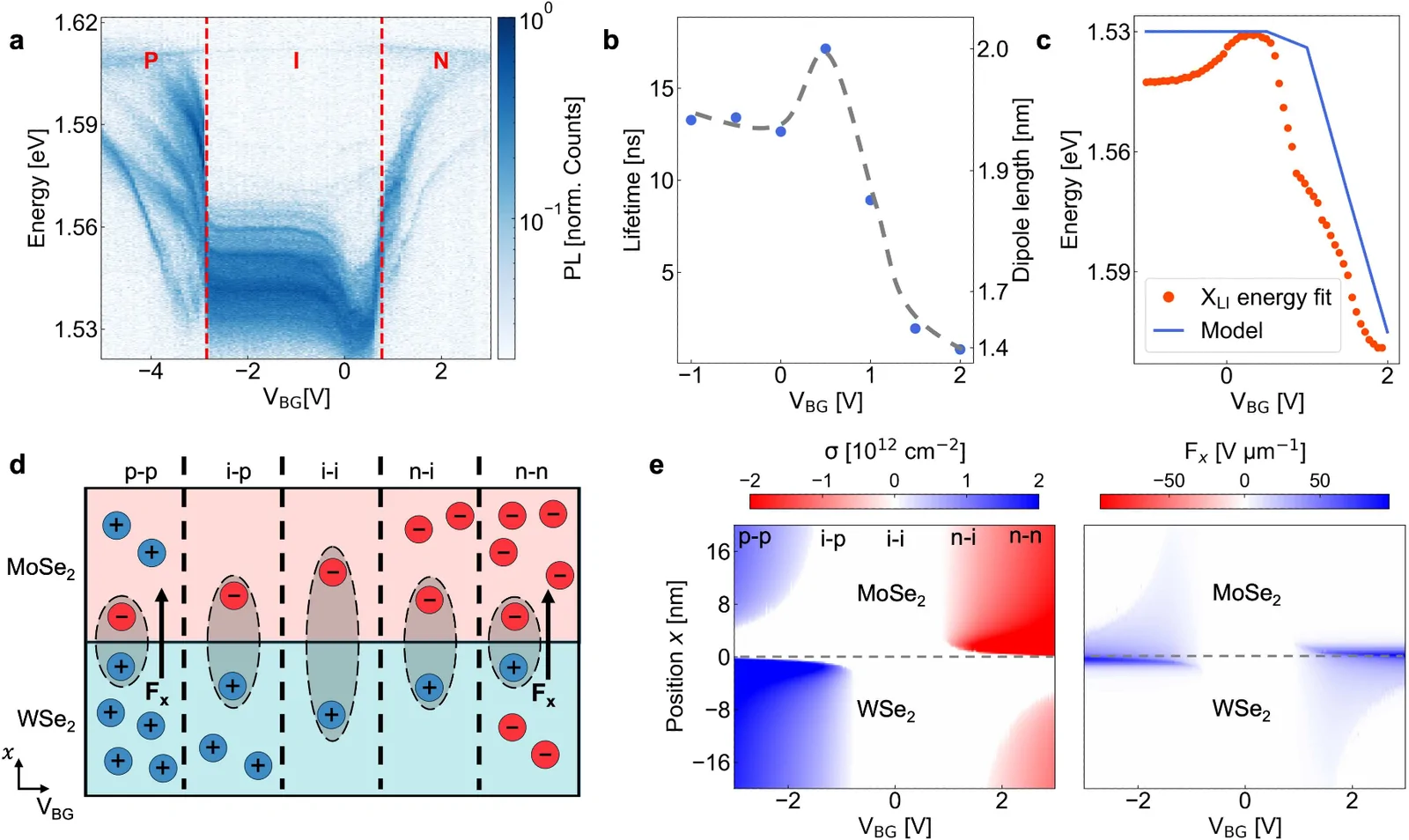
Dynamic Control over Dipole Moment and Lifetime via Electrostatic Doping. **a**, PL spectra as a function of the bottom gate voltage (*V*_BG_) show a large, systematic blue shift (∼70meV) of the *X*_LI_ state upon charge doping. **b**, Measured *X*_LI_ lifetime (*τ*, blue circles, left axis) on the n-doping side reduces by more than an order of magnitude from 15 ns at neutrality to 800 ps at *V*_BG_ = 2 V. The corresponding dipole length (*d*_e‑h_) extracted from *τ* (right axis) demonstrates dynamic tunability from 2 to 1.4 nm. **c**, Extracted *X*_LI_ center energy (orange points) as a function of *V*_BG_. The blue curve represents the computed energy based on the DC Stark shift model, using the dipole lengths extracted independently from the lifetime data in (**b**) and simulated electric fields (**e**). The excellent qualitative agreement confirms the model linking lifetime, dipole collapse, and spectral energy. **d**, Schematic illustrating the Type-II alignment under doping: charge accumulation generates an in-plane electric field (*F*_*x*_) across the interface that opposes the permanent dipole moment (p⃗) of the exciton. **e**, Finite-element simulations confirming the concentration of strong in-plane electric fields near the interface upon doping, which drives the dipole collapse.

To understand the origin of this shift, we measure the *X*_LI_ lifetime using TCSPC (Figure 4b) as a function of *V*_BG_. We observe a dramatic reduction in the lifetime by more than an order-of-magnitude, dropping from *τ* ≈ 15 ns at neutrality to *τ* ≈ 800 ps at *V*_BG_ = 2 V. Crucially, this lifetime collapse occurs in synchrony with the observed blue shift (Figure 4c), suggesting that both observables probe a common underlying reconfiguration of the exciton’s internal structure.

We propose that this behavior is the direct result of a gate-controlled “dipole collapse.” This effect can be viewed through two complementary yet equivalent lenses: first, gate-induced doping generates a concentrated in-plane electric field *F*_*x*_ that is anti-aligned to the dipole regardless of the doping configuration (see Figure 4d). This field, confirmed by electrostatic simulations, shown in Figure 4e, exerts a compressive force on the dipolar exciton. Alternatively, the onset of doping screens and renormalizes the intrinsic band-offset, effectively flattening the Type-II potential. This screening reduces the electrostatic barrier that partitions the carriers, allowing the electron and hole wavefunctions to penetrate more deeply into the adjacent regions, thereby increasing their spatial overlap.

To quantify this structural change, we utilize a tunneling model^17^ where the electron and hole wavefunctions are treated as quasi-bound states with evanescent tails: $\psi_{e,h}∼\exp\left(\frac{-\left|x\pm d_{e‐h}/2\right|}{b}\right)$ , where *b* is a tunneling length (see SI for further details). The electron-hole wavefunction overlap relates directly to the dipole length, according to $O_{eh}=\int \psi_{e}^{*}\psi_{h}dx\propto \exp\left(\frac{-d_{e‐h}}{b}\right)$ . Hence, enhancement of the overlap reduces radiative lifetime $\left(\tau \propto O_{e‐h}^{-2}\right)$ , through the relation: $\tau =\tau_{0}\exp\left(\frac{2d_{e‐h}}{b}\right)$ , where *τ*_0_ ≈ 1 ps is the known homogeneously broadened radiative lifetime of a 1s exciton with maximal overlap (*d*_e‑h_ = 0).^33^ By anchoring this model to our measured reference values of dipole length ${\mathrm{d̃}}_{e‐h}=2\mathrm{nm}$ and lifetime τ̃=15ns at neutrality, we derive a zero-parameter relationship to extract the *V*_BG_-dependent dipole length:

1 $$d_{e‐h}\left(V_{BG}\right)={\mathrm{d̃}}_{e‐h}\frac{\ln\left(\tau \left(V_{BG}\right)/\tau_{0}\right)}{\ln\left(\mathrm{\tau ̃}/\tau_{0}\right)}$$

For simplicity, this model assumes a constant tunneling length *b*. While *b* may physically vary as the gate-dependent potential renormalization modifies the barrier height, we find that the modulation of the dipole length *d*_e‑h_ is the primary driver of the observed dynamics. The extracted dipole lengths (Figure 4b, right axis) reveal a dynamic tuning of the exciton’s internal size from 2 nm down to ∼1.4nm . To validate this model, we calculate the energy shift, $\Delta E\left(V_{BG}\right)=-e{\mathrm{d⃗}}_{e‐h}\left(V_{BG}\right)\cdot {\mathrm{F⃗}}_{x,\max}\left(V_{BG}\right)$ , using only these independently extracted dipole lengths and simulated maximum field amplitude at the interface (Figure 4e). The resulting curve (Figure 4c, blue line) shows remarkable consistency with the experimental data.

We note that the measured lifetime (*τ*_meas_) includes both radiative (*τ*_rad_) and non-radiative (*τ*_nr_) contributions, according to 1/*τ*_meas_ = 1/*τ*_rad_ + 1/*τ*_nr_. In addition, we expect significant inhomogeneous broadening of the interfacial excitonic state, which likely leads to the enhanced line width observed in our experiments. However, non-radiative channels alone would not typically produce a sharp lifetime collapse in synchrony with a monotonic blue shift. The fact that a structural change in the excitonic wavefunction, inferred solely from temporal dynamics, predicts the order-of-magnitude energy shift provides definitive evidence for the dipole collapse mechanism, and justifies the exclusion of non-radiative effects from the heuristic tunneling model.^43^ A detailed study of non-radiative contributions to the interfacial exciton dynamics is the subject of future work. This result establishes the lateral interface as a unique platform where the internal quantum structure of an exciton can be continuously engineered in-situ.

Our findings establish the lateral MoSe_2_/WSe_2_ interface as an intrinsic 1D quantum wire, where excitonic quasiparticles emerge via crystallographic control and electron-hole interactions rather than top-down lithography.

The lateral architecture enables in-situ electrostatic tuning of the exciton’s internal dipole and radiative rate, in contrast to vertical heterostructures where interlayer exciton properties are fixed by layer separation. Beyond its structural tunability, this interface quantum wire represents an interesting experimental path serving as proof of concept for excitonic technologies. The high mobilities $\left(∼1200{\mathrm{cm}}^{2}/\mathrm{Vs}\right)$ suggest that interfacial excitons are protected by suppressed momentum relaxation due to reduced dimensionality. Such high mobility positions these atomically sharp channels as low-loss interconnects. Moreover, the strong lateral confinement and large dipole moment of the interfacial excitons make these 1D junctions attractive gate-tunable channels for excitonic transistors, extending the switching concepts already demonstrated in lateral TMD heterojunction devices^44^ toward operation at the single-exciton level.

The strict 1D localization of *X*_LI_, together with its pronounced sensitivity to the local charge environment at the interface, suggests its utility as a non-invasive probe of electronic order at the edges of two-dimensional semiconductors. By residing exclusively at the junction and directly coupling to charge distributions along the boundary, the interfacial exciton provides a nanometer-scale optical sensor of edge states and electronic fluctuations—phenomena that are largely inaccessible to conventional bulk optical measurements.

Finally, the large permanent dipole moment and stable 1D confinement provide an ideal arena for many-body physics.^45^ The strong repulsive interactions and independent control over confinement and density offer a unique tuning parameter to realize strongly correlated phases, such as dipolar crystals, moving well beyond the dilute weakly interacting limit. In summary, this lateral junction provides a unique platform for the active engineering of 1D excitonic devices and the exploration of novel quantum phases with reduced dimensions.

## Supplementary Material



## Notes

Experimental setup, device fabrication and characterization, lateral interface exciton PL noise analysis and time trace, theoretical treatment of interfacial exciton dynamics, diffusion measurements, theoretical framework for 1D exciton diffusion, field-dependent and doping-dependent measurements, lifetime and power measurements, electrostatic simulation of LHS device, data analysis procedure, dipole moment uncertainty, and distinguishing interfacial excitons from defect-bound states (PDF)

## References

[1] Cazalilla M. A., Citro R., Giamarchi T., Orignac E., Rigol M. (2011). One dimensional
bosons: From condensed matter systems to ultracold gases. Rev. Mod. Phys..

[2] Ołdziejewski R., Chiocchetta A., Knörzer J., Schmidt R. (2022). Excitonic Tonks-Girardeau
and charge density wave phases in monolayer semiconductors. Phys. Rev. B.

[3] Sakaki H. (1980). Scattering
suppression and high-mobility effect of size-quantized electrons in
ultrafine semiconductor wire structures. Jpn.
J. Appl. Phys..

[4] van
Wees B. J., van Houten H., Beenakker C. W. J., Williamson J. G., Kouwenhoven L. P., van der Marel D., Foxon C. T. (1988). Quantized conductance of point contacts
in a two-dimensional electron gas. Phys. Rev.
Lett..

[5] Kane B. E., Facer G. R., Dzurak A. S., Lumpkin N. E., Clark R. G., Pfeiffer L. N., West K. W. (1998). Quantized
conductance in quantum
wires with gate-controlled width and electron density. Appl. Phys. Lett..

[6] Colombo C., Spirkoska D., Frimmer M., Abstreiter G., Fontcuberta i Morral A. (2008). Ga-assisted
catalyst-free growth mechanism of GaAs
nanowires by molecular beam epitaxy. Phys. Rev.
B.

[7] Li Y., Hu Z., Guo Q., Li J., Liu S., Xie X., Zhang X., Kang L., Li Q. (2025). van der Waals one-dimensional
atomic crystal heterostructure derived from carbon nanotubes. Chem. Soc. Rev..

[8] Huang C., Wu S., Sanchez A. M., Peters J. J. P., Beanland R., Ross J. S., Rivera P., Yao W., Cobden D. H., Xu X. (2014). Lateral heterojunctions
within monolayer MoSe_2_-WSe_2_ semiconductors. Nat. Mater..

[9] Duan X., Wang C., Shaw J. C., Cheng R., Chen Y., Li H., Wu X., Tang Y., Zhang Q., Pan A., Jiang J., Yu R., Huang Y., Duan X. (2014). Lateral epitaxial
growth of two-dimensional layered semiconductor heterojunctions. Nat. Nanotechnol..

[10] Li M.-Y., Shi Y., Cheng C.-C., Lu L.-S., Lin Y.-C., Tang H.-L., Tsai M.-L., Chu C.-W., Wei K.-H., He J.-H., Chang W.-H., Suenaga K., Li L.-J. (2015). Epitaxial growth
of a monolayer WSe_2_-MoS_2_ lateral *p-n* junction with an atomically sharp interface. Science.

[11] Sahoo P. K., Memaran S., Xin Y., Balicas L., Gutiérrez H. R. (2018). One-pot
growth of two-dimensional lateral heterostructures via sequential
edge-epitaxy. Nature.

[12] Wang G., Chernikov A., Glazov M. M., Heinz T. F., Marie X., Amand T., Urbaszek B. (2018). Colloquium: Excitons in atomically
thin transition metal dichalcogenides. Rev.
Mod. Phys..

[13] Thureja D., Imamoglu A., Smoleński T., Amelio I., Popert A., Chervy T., Lu X., Liu S., Barmak K., Watanabe K., Taniguchi T., Norris D. J., Kroner M., Murthy P. A. (2022). Electrically tunable
quantum confinement of neutral
excitons. Nature.

[14] Dirnberger F., Ziegler J. D., Faria Junior P. E., Bushati R., Taniguchi T., Watanabe K., Fabian J., Bougeard D., Chernikov A., Menon V. M. (2021). Quasi-1d exciton
channels in strain-engineered 2d materials. Sci. Adv..

[15] Degani M. H., Farias G. A. (1990). Exciton binding
energy in type-ii heterojunctions. Phys. Rev.
B.

[16] Kang J., Tongay S., Zhou J., Li J., Wu J. (2013). Band offsets
and heterostructures of two-dimensional semiconductors. Appl. Phys. Lett..

[17] Lau K. W., Calvin, Gong Z., Yu H., Yao W. (2018). Interface excitons at lateral heterojunctions in monolayer
semiconductors. Phys. Rev. B.

[18] Wilson N. R., Nguyen P. V., Seyler K., Rivera P., Marsden A. J., Laker Z. P. L., Constantinescu G. C., Kandyba V., Barinov A., Hine N. D. M., Xu X., Cobden D. H. (2017). Determination of
band offsets, hybridization, and exciton binding in 2d semiconductor
heterostructures. Sci. Adv..

[19] Rosati R., Paradisanos I., Huang L., Gan Z., George A., Watanabe K., Taniguchi T., Lombez L., Renucci P., Turchanin A., Urbaszek B., Malic E. (2023). Interface engineering
of charge-transfer excitons in 2d lateral heterostructures. Nat. Commun..

[20] Lima I. L. C., Milošević M. V., Peeters F. M., Chaves A. (2023). Tuning of
exciton type by environmental screening. Phys.
Rev. B.

[21] Durnev M. V., Smirnov D. S. (2025). Intervalley mixing
of interface excitons at lateral
heterojunctions. Phys. Rev. B.

[22] Rivera P., Schaibley J. R., Jones A. M., Ross J. S., Wu S., Aivazian G., Klement P., Seyler K., Clark G., Ghimire N. J., Yan J., Mandrus D. G., Yao W., Xu X. (2015). Observation of long-lived
interlayer excitons in monolayer MoSe_2_-WSe_2_ heterostructures. Nat.
Commun..

[23] Guo Y., Robertson J. (2016). Band engineering
in transition metal dichalcogenides:
Stacked versus lateral heterostructures. Appl.
Phys. Lett..

[24] Jiang Y., Chen S., Zheng W., Zheng B., Pan A. (2021). Interlayer
exciton formation, relaxation, and transport in TMD van der Waals
heterostructures. Light Sci. Appl..

[25] Yuan L., Zheng B., Zhao Q., Kempt R., Brumme T., Kuc A. B., Ma C., Deng S., Pan A., Huang L. (2023). Strong dipolar repulsion
of one-dimensional interfacial excitons
in monolayer lateral heterojunctions. ACS Nano.

[26] Beret D., Paradisanos I., Lamsaadi H., Gan Z., Najafidehaghani E., George A., Lehnert T., Biskupek J., Kaiser U., Shree S., Estrada-Real A., Lagarde D., Marie X., Renucci P., Watanabe K., Taniguchi T., Weber S., Paillard V., Lombez L., Poumirol J.-M., Turchanin A., Urbaszek B. (2022). Exciton spectroscopy and unidirectional
transport in MoSe_2_-WSe_2_ lateral heterostructures
encapsulated in hexagonal boron nitride. npj
2D Mater. Appl..

[27] Kundu B., Mondal P., Tebbe D., Hasan M. N., Chakraborty S. K., Metzelaars M., Kögerler P., Karmakar D., Pradhan G. K., Stampfer C., Beschoten B., Waldecker L., Sahoo P. K. (2024). Electrically controlled
excitons, charge transfer induced
trions, and narrowband emitters in MoSe_2_-WSe_2_ lateral heterostructure. Nano Lett..

[28] Rosati R., Shradha S., Picker J., Turchanin A., Urbaszek B., Malic E. (2025). Impact of charge-transfer
excitons
on unidirectional exciton transport in lateral TMD heterostructures. Nano Lett..

[29] Chu Y.-H., Wang L.-H., Lee S.-Y., Chen H.-J., Yang P.-Y., Butler C. J., Lu L.-S., Yeh H., Chang W.-H., Lin M.-T. (2018). Atomic scale depletion region at
one dimensional MoSe_2_-WSe_2_ heterointerface. Appl.
Phys. Lett..

[30] Srivastava A., Sidler M., Allain A. V., Lembke D. S., Kis A., Imamoğlu A. (2015). Optically
active quantum dots in monolayer WSe_2_. Nat. Nanotechnol..

[31] Chakraborty C., Goodfellow K. M., Vamivakas A. N. (2016). Localized emission from defects in
MoSe_2_ layers. Opt. Mater. Express.

[32] Lorchat E., López L. E. P., Robert C., Lagarde D., Froehlicher G., Taniguchi T., Watanabe K., Marie X., Berciaud S. (2020). Filtering
the photoluminescence spectra of atomically thin semiconductors with
graphene. Nat. Nanotechnol..

[33] Robert C., Lagarde D., Cadiz F., Wang G., Lassagne B., Amand T., Balocchi A., Renucci P., Tongay S., Urbaszek B., Marie X. (2016). Exciton radiative lifetime
in transition
metal dichalcogenide monolayers. Phys. Rev.
B.

[34] Kumar N., Cui Q., Ceballos F., He D., Wang Y., Zhao H. (2014). Exciton diffusion
in monolayer and bulk MoSe_2_. Nanoscale.

[35] Sun Z., Ciarrocchi A., Tagarelli F., Gonzalez Marin J. F., Watanabe K., Taniguchi T., Kis A. (2022). Excitonic transport
driven by repulsive dipolar interaction in a van der Waals heterostructure. Nat. Photonics.

[36] Wietek E., Florian M., Göser J., Taniguchi T., Watanabe K., Högele A., Glazov M. M., Steinhoff A., Chernikov A. (2024). Nonlinear and negative effective diffusivity of interlayer
excitons in moiré-free heterobilayers. Phys. Rev. Lett..

[37] Crochet J. J., Duque J. G., Werner J. H., Lounis B., Cognet L., Doorn S. K. (2012). Disorder limited exciton transport in colloidal single-wall
carbon nanotubes. Nano Lett..

[38] Li Z., Florian M., Datta K., Jiang Z., Borsch M., Wen Q., Kira M., Deotare P. B. (2023). Enhanced exciton drift transport
through suppressed diffusion in one-dimensional guides. ACS Nano.

[39] Wu D., Li X., Luan L., Wu X., Li W., Yogeesh M. N., Ghosh R., Chu Z., Akinwande D., Niu Q., Lai K. (2016). Uncovering edge states
and electrical inhomogeneity
in MoS_2_ field-effect transistors. Proc. Natl. Acad. Sci. U.S.A..

[40] Moore D., Jo K., Nguyen C., Lou J., Muratore C., Jariwala D., Glavin N. R. (2020). Uncovering topographically hidden features in 2d MoSe_2_ with correlated potential and optical nanoprobes. npj 2D Mater. Appl..

[41] Hu J., Lorchat E., Chen X., Watanabe K., Taniguchi T., Heinz T. F., Murthy P. A., Chervy T. (2024). Quantum control of
exciton wave functions in 2d semiconductors. Sci. Adv..

[42] Szafran B. (2022). Exciton localization
on *p* – *i* – *n* junctions in two-dimensional crystals. Phys. Rev. B.

[43] Jauregui L. A., Joe A. Y., Pistunova K., Wild D. S., High A. A., Zhou Y., Scuri G., Greve K. D., Sushko A., Yu C.-H., Taniguchi T., Watanabe K., Needleman D. J., Lukin M. D., Park H., Kim P. (2019). Electrical control
of interlayer exciton dynamics in atomically thin heterostructures. Science.

[44] Kundu B., Chakrabarty P., Dhara A., Rosati R., Samanta C., Chakraborty S. K., Sahoo S., Dash S. P., Dhara S., Malic E., Lodha S., Sahoo P. K. (2026). Trion-engineered
multimodal photo-transistors in two-dimensional lateral heterostructures. Adv. Funct. Mater..

[45] Vandoolaeghe, E. , Photon correlation microscopy of quantum matter (accessed May 27th 2026), arXiv:2605.28623 [cond-mat.mes-hall].

