# Supplementary material for: Dipolar Excitonic Quantum Wires at Atomically Sharp Lateral Interfaces
Source: Nano Lett. 2026 Sep 15;26(44):15215–22. doi: 10.1021/acs.nanolett.6c02624 (PMC13761681; doi:10.1021/acs.nanolett.6c02624)
Supplement: Supplementary file 1 [file nl6c02624_si_001.pdf]

Supplementary information of "Dipolar Excitonic Quantum Wires at Atomically Sharp Lateral Interfaces"

Elie Vandoolaeghe[1], Francesco Fortuna[1], Suman Kumar Chakraborty[2], Biswajeet Nayak[2], Takashi Taniguchi[3], Kenji Watanabe[3], Prasana Kumar Sahoo[2], Thibault Chervy[4], and Puneet A. Murthy[1*]

July 23, 2026

[1]Institute for Quantum Electronics, ETH Zürich, CH-8093 Zürich, Switzerland
[2]Quantum Materials and Device Research Lab, Materials Research Center, Indian Institute of Technology, Karagpur, India
[3]Q. National Institute for Materials Science, Namiki 1-1, Tsukuba, 305-0044, Ibaraki, Japan
[4]NTT Research, Inc. Physics & Informatics Laboratories, 940 Stewart Dr, Sunnyvale, CA 94085

1 Experimental Setup

We describe the experimental setup and general measurement conditions used to produce the data shown in this text.

All measurements are conducted at cryogenic temperatures using a fiber-channeled optical setup that also provides electrical access to the sample. Supplementary Information Fig. 1 presents a schematic overview of the setup. The imaging path (highlighted in yellow) employs a white-light LED for real-time visualization of the sample. The excitation source (highlighted in red) is transmitted through a single-mode fiber (SMF) and its power is controlled using an electronic variable optical attenuator (VOA). The input light is divided by a 50:50 beam splitter (BS); one half is directed to the sample, while the other half is directed to a power monitoring branch. The excitation light reaching the sample is focused by a high numerical aperture (NA = 0.7) objective, resulting in a beam diameter of approximately 700 nm. Emission from the sample is subsequently collected and sent through a single-mode fiber to a spectrometer.

The sample is mounted on a printed circuit board (PCB), which serves as the interface for all electrical connections. Bias voltages are supplied to the device via the PCB, and wire bonding

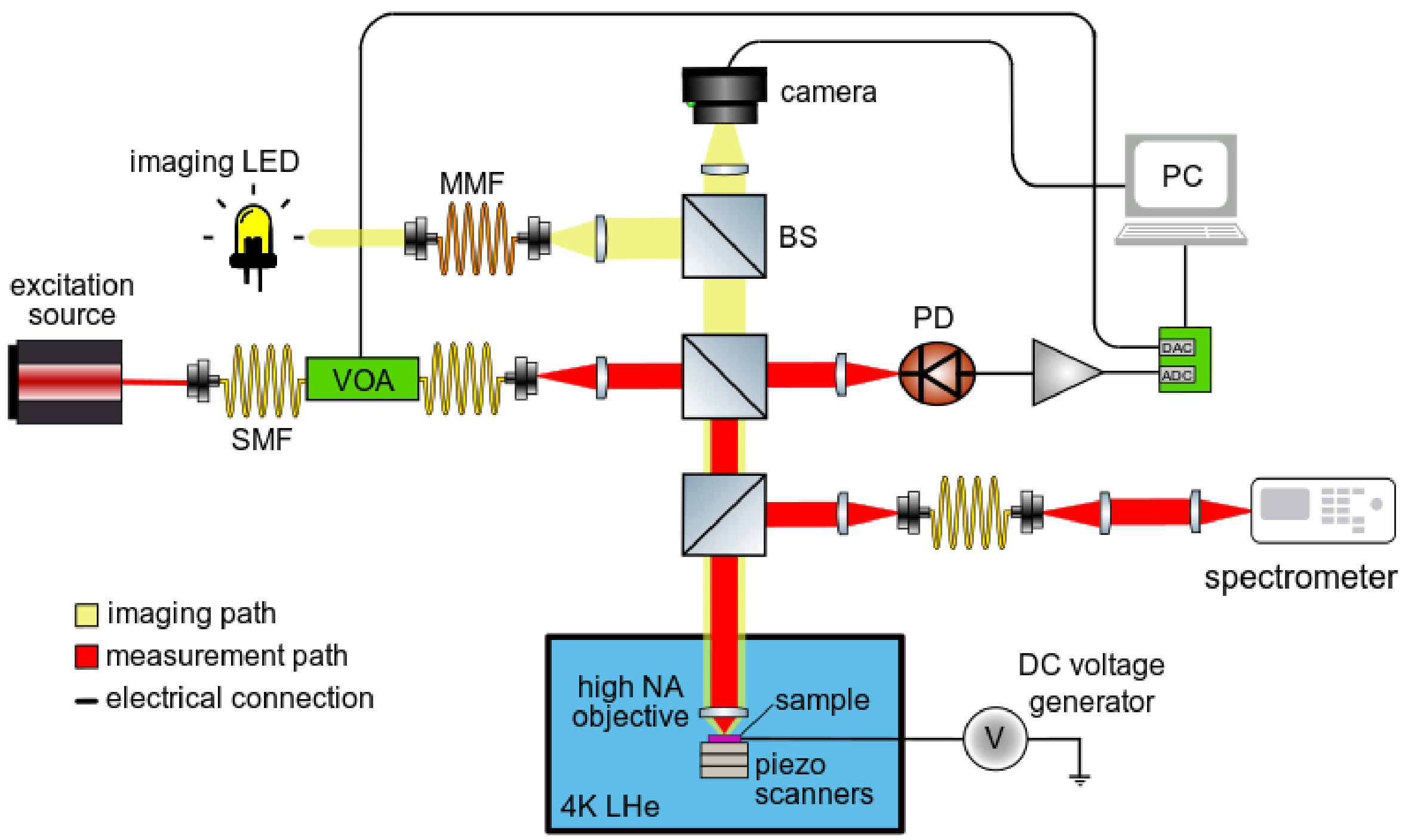

Figure 1: **Confocal microscopy setup**. The setup features an imaging path, which uses a white-light LED to provide real-time visualization of the sample, and a measurement path, supplying excitation light through a single-mode fiber. The excitation beam is split by a 50:50 beam splitter, with one branch directed to the sample via a high-NA objective and the other monitored by a photodiode for power control. Emission from the sample is collected by the same objective and sent to a spectrometer. The sample is mounted on a PCB for electrical biasing, which in turn is placed on top of piezoelectric nanopositioners for precise X-Y-Z alignment. The sample assembly is housed in a liquid helium dewar to maintain cryogenic temperatures.

is used to connect the chip's electrode pads to the PCB circuitry. The PCB is secured to a stack of X-Y-Z piezoelectric nanopositioners that allow for detailed position scans and fine focus adjustments. The entire assembly— sample, PCB, and nanopositioners—is housed within a stainless steel tube, filled with 20 mbar helium exchange gas, which is then inserted into a liquid helium dewar.

2 Device Fabrication and Characterization

We give the basic details of the classic TMDCs heterostructure sample fabrication, as well as some details about the grown flakes used. We describe the different types of samples obtained and the data reproducibility.

Samples are stacked using a dry stacking procedure with PDMS droplets covered by a PC film. The LHS flakes are grown on SiO_2 substrates and are picked up, alongside the other graphene and encapsulating flakes, by a top hBN. We contact the samples using prepatterned gold electrodes on Si/SiO_2 substrates. After the stack is assembled, 13 nm-thick gold split gates are deposited on top using e-beam lithography, e-beam evaporation of gold and bilayer liftoff.

We identify two types of lateral interfaces in our CVD-grown $MoSe_2$/WSe_2 heterostructures in the outwards direction:

WSe_2 $\rightarrow$$MoSe_2$ interfaces, which display an atomically sharp transition, and $MoSe_2$ $\rightarrow$$WSe_2$ interfaces, which exhibit a smooth transition extending over approximately 5–10 nm with some degree of diffuse replacement of one atomic species into the other [1]. While a broader interface increase the excitonic dipole length, we expect that it significantly reduces the electron–hole wavefunction overlap, leading to a much lower radiative recombination rate and thus a strongly suppressed photoluminescence (PL) signal.

Devices 1–3 share the same multi-interface geometry: a large triangular $MoSe_2$ domain flanked by two ~ 1 µm wide WSe_2 strips, with a narrow (~ 50 nm) $MoSe_2$ channel embedded between them (Supplementary Information Fig. 2). This configuration contains two smooth interfaces (W grown from Mo) and one sharp interface (Mo grown from W). Results from Device 1 are shown in the main text, while Devices 2 and 3 are included here for consistency and spatially resolved PL analysis.

To directly compare the emission behavior of sharp and smooth interfaces, we fabricate Device 4, which features a simpler structure: a triangular $MoSe_2$ region flanked by a single WSe_2 strip, forming only a smooth interface.

Low-temperature (4 K) PL spectra from Devices 1–3 display a bright lateral interface exciton (X_{LI}) peak approximately 100 meV below the $MoSe_2$ neutral exciton (X_{Mo}), together with the usual intralayer exciton and trion features (Supplementary Information Fig. 3a–b). In Device 4, however, the X_{LI} emission is suppressed by a factor of $\sim 10^5$ compared to Device 1–3. This estimation accounts for differences in sample brightness (by normalizing each spectrum to the $MoSe_2$ intralayer exciton PL intensity), excitation power and integration time (Supplementary Information Fig. 3c). Device 4 confirms that the smooth interface does not contribute to the

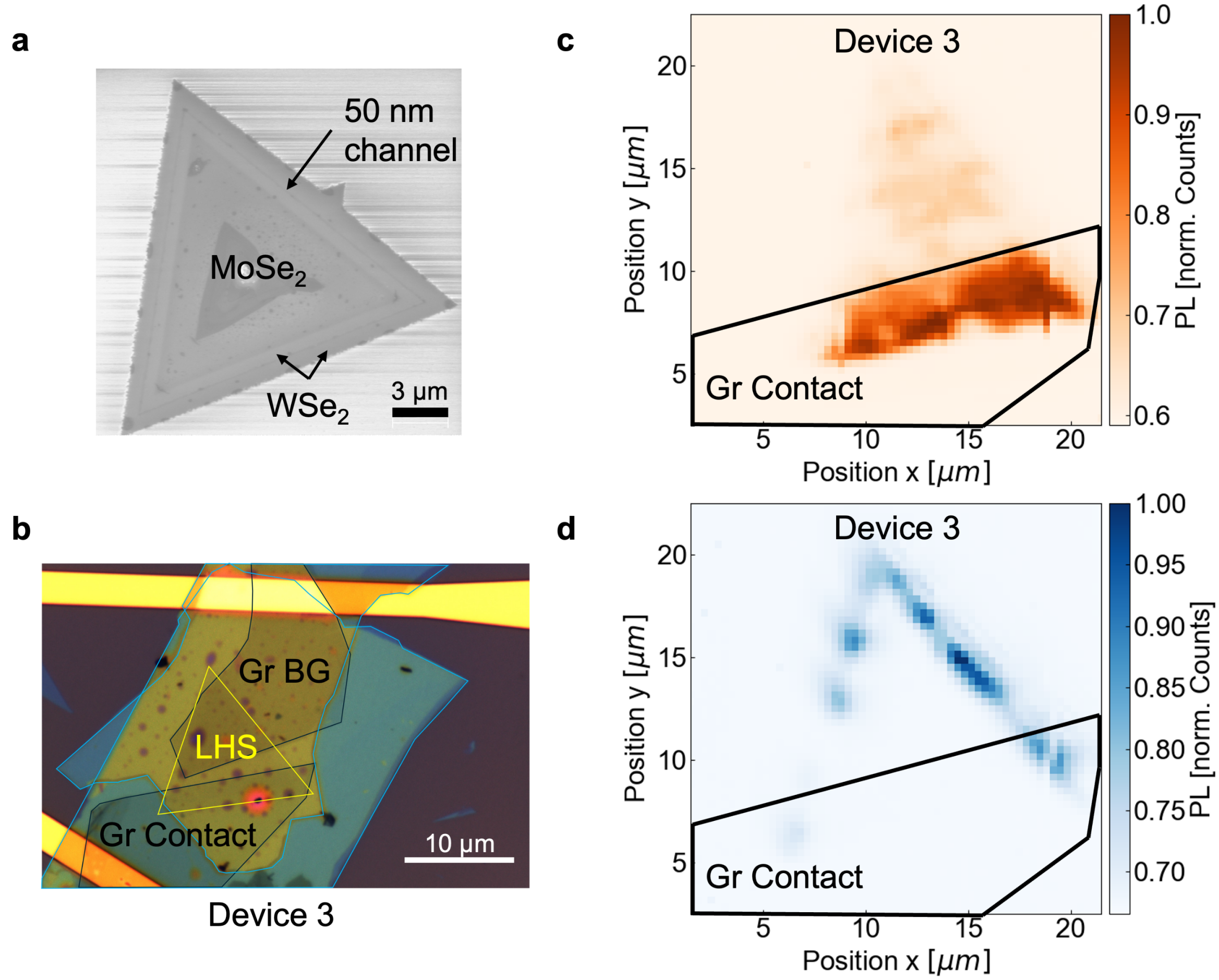

Figure 2: **LHS material composition**. **a** Scanning Electron Microscopy (SEM) image of a CVD-grown lateral heterostructure (LHS) of monolayer $MoSe_2$ and WSe_2 used for devices 1-3. The structure features a large central triangular domain of $MoSe_2$, flanked by two ~ 1µm wide WSe_2 strips. A narrow ~ 50 nm $MoSe_2$ channel runs between the two WSe_2 regions. **b** Micrograph of device 3 after fabrication steps. **c** Spatially resolved PL scan integrated over $MoSe_2$ states across the device, showcasing the sample homogeneity. **d** Spatially resolved PL scan integrated over X_{LI} state across the device reveals that the new state is exclusively emitted at the 1D interface. The emission at this energy is observed across nearly the entire active region of the device, spanning about 30 microns.

$X_{\rm LI}$ signal under the experimental conditions presented in the main text and allows us to unambiguously distinguish the optical response of sharp versus smooth interface, confirming that all presented $X_{\rm LI}$ emission originates from sharp junctions.

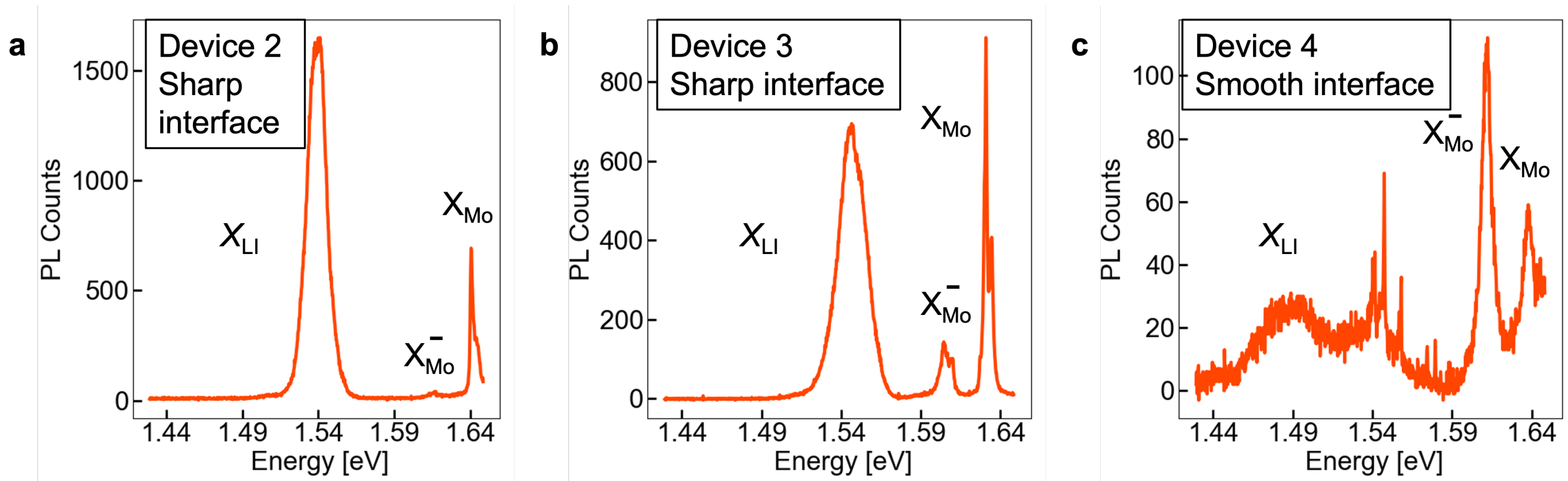

Figure 3: **Single spectra from different interfaces and devices**. **a, b** Photoluminescence (PL) spectra measured at 4 K, obtained from the sharp interface in devices 2 and 3. At the interface, in addition to the typical exciton and trion states of $MoSe_2$ and WSe_2 ($X_{\rm Mo}$, $X_{\rm W}$) on either side, a new bright state is observed approximately 100 meV below the neutral direct exciton. **c,** PL spectra measured at 4 K from the smooth interface in Device 4. The $X_{\rm LI}$ emission at the smooth interface is significantly suppressed, approximately 10^5 times dimmer, compared to the sharp interface. This estimation accounts for differences in sample brightness (by normalizing each spectrum to the $MoSe_2$ intralayer exciton PL intensity), excitation power and integration time.

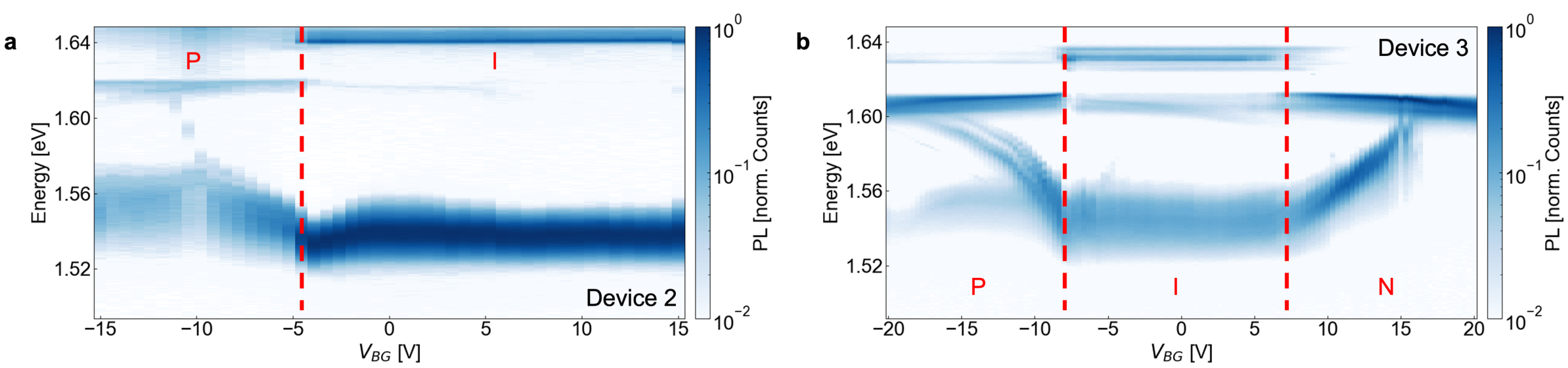

Figure 4: **Doping-induced dispersion in Device 2 and 3**. **a, b** PL spectra as a function of $V_{\rm BG}$ in devices 2 and 3 show a blue shift of the lateral interface exciton with doping. Inconsistencies of doping are due to contacting and intrinsic effects.

Spatially resolved PL maps (Supplementary Information Fig. 2d) further show that the $X_{\rm LI}$ emission in Device 3 is localized exclusively at the interface and extends uniformly over ~ 30 µm.

Finally, doping-induced dispersion in Devices 2 and 3 is presented in Supplementary Information Fig. 4. PL spectra as a function of $V_{\rm BG}$ in Devices 2 and 3 exhibit a strong blue shift of

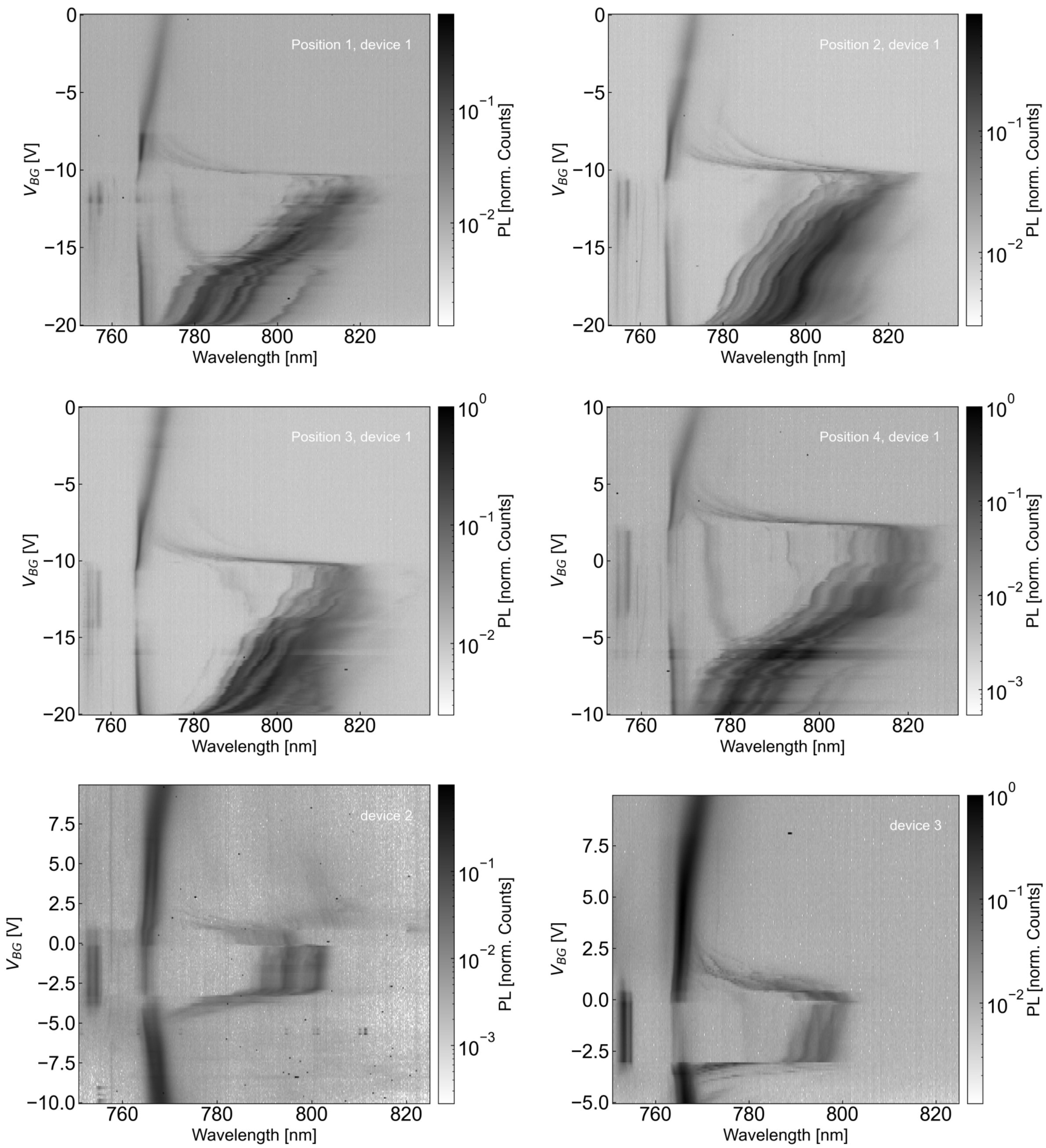

Figure 5: **Raw spectra versus back gate voltage taken at low power, showing COM quantization**. PL spectra as a function of position in device 1 and in a single position in devices 2 and 3 show the ladder of COM-confined states, with slight differences in the spectral structure.

the lateral interface exciton with doping, consistent with Device 1 behavior. Inconsistencies of doping are due to contacting and intrinsic effects.

3 Lateral Interface Exciton PL Noise Analysis and Time Trace

We discuss in this section the difference between lateral interface excitonic state and some other possible states such as growth defects that could be located at the interface and exhibiting PL. We justify the spectra to be assigned to the lateral interface excitons.
The LI-exciton spectra in Fig. 3c exhibit several discrete emission lines whose fluctuations are correlated across all peaks, indicating a single, common excitonic origin rather than multiple independent defect centers. Moreover, defect-bound emitters in monolayer TMDs often show random on/off switching and slow spectral drift: for example, monolayer MoS_2 quantum emitters undergo telegraph-noise blinking due to stochastic charge trapping and detrapping at localized defect sites [2], and WS_2 defect-bound excitons display slow PL spectral wandering arising from charge fluctuations in the emitter environment [3]. However, the time-resolved PL trace in Supplementary Information Fig. 6, obtained with 30 s integration time under zero gate voltage, remains essentially noise-free and spectrally invariant. The absence of any blinking or spectral diffusion over this period confirms the intrinsic, non-defect nature of the LI exciton emission.

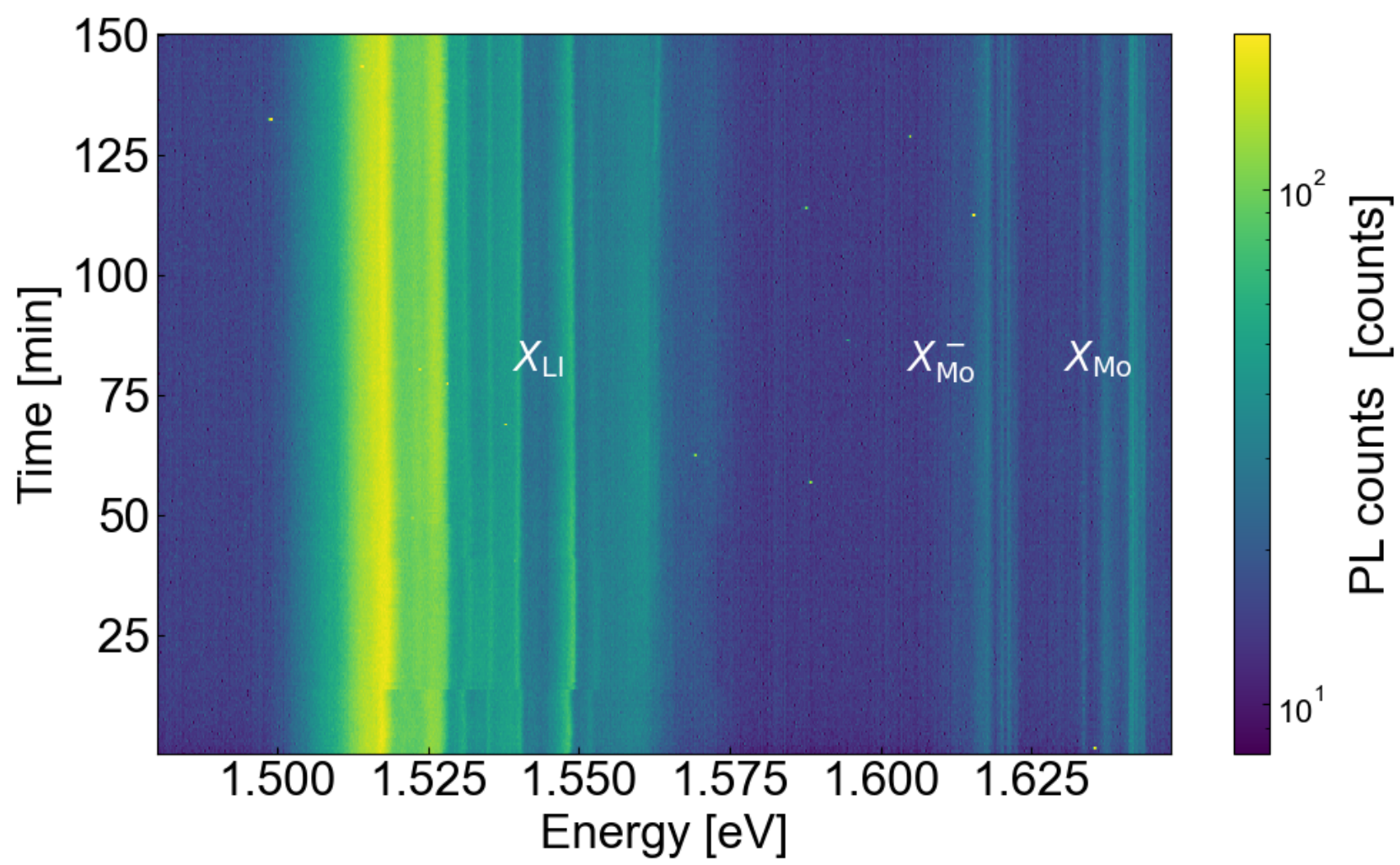

Figure 6: **PL spectra time trace.** PL spectra measured over 150 minutes. The LI exciton signal is stable and does not exhibit significant blinking behavior. Each spectrum is integrated for 30 seconds.

4 Theoretical Treatment of Interfacial Exciton Dynamics

This section details the theoretical framework for the 1D interfacial exciton (X_{LI}) emerging at the lateral heterojunction. We describe the separation of center-of-mass and relative degrees of freedom, the nature of the 1D confinement, and the scaling laws governing the radiative and non-radiative decay dynamics.

4.1 System Hamiltonian and Coordinate Transformation

We describe the electron-hole pair at the lateral $\mathrm{MoSe_2/WSe_2}$ junction within the effective mass approximation. The Hamiltonian is given by:

$$H = -\frac{\hbar^2}{2m_e^*}\nabla_e^2 - \frac{\hbar^2}{2m_h^*}\nabla_h^2 + V_{\text{ext}}(x_e, x_h) + V_C(\mathbf{r}_e, \mathbf{r}_h) \tag{1}$$

where m_e^* and m_h^* are the effective masses. V_{ext} represents the lateral interface potential defined by the Type-II band offsets, and V_C is the screened Rytova-Keldysh Coulomb potential. To analyze the 1D nature of the system, we transform to center-of-mass ($\mathbf{R}$) and relative ($\mathbf{r}$) coordinates:

$$\mathbf{R} = \frac{m_e^*\mathbf{r}_e + m_h^*\mathbf{r}_h}{M}, \quad \mathbf{r} = \mathbf{r}_e - \mathbf{r}_h \tag{2}$$

where $M = m_e^* + m_h^*$ is the total mass. For an interface aligned along the y-axis, the total wavefunction $\Psi(\mathbf{R}, \mathbf{r})$ is expressed as the product of the COM wavefunction $\Xi(X, Y)$ and the internal relative wavefunction $\phi(x, y)$.

4.2 1D COM Confinement and the Quantized Ladder

The transverse motion of the COM (X) is restricted by an effective potential $V_{\text{conf}}(X)$ resulting from the junction's band alignment and the local electrostatic environment. This leads to the 1D Schrödinger equation for the COM:

$$\left[-\frac{\hbar^2}{2M}\frac{\partial^2}{\partial X^2} + V_{\text{conf}}(X)\right]\Xi_n(X) = E_{n,\text{COM}}\Xi_n(X) \tag{3}$$

Treating $V_{\text{conf}}(X)$ as a parabolic or V-shaped potential well, we obtain a discrete ladder of states $E_{n,\text{COM}}$. Each peak observed in the low-energy PL spectrum corresponds to a different quantized vibrational mode of the exciton's center-of-mass within the 1D wire.

4.3 Relative Wavefunctions and the Tunneling Length b

The relative wavefunction $\phi(x, y)$ describes the internal dipolar structure. The Type-II band offset separates the electron and hole by an average distance $d_{\text{e-h}}$. In the transverse relative

coordinate x, the wavefunctions are modeled as quasi-bound states with evanescent tails penetrating the junction barrier:

$$\phi(x) \propto \exp\left(-\frac{|x \mp d_{\text{e-h}}/2|}{b}\right) \tag{4}$$

The tunneling length b characterizes the spatial extent of the wavefunction penetration and is determined by:

$$b = \frac{\hbar}{\sqrt{2m^* \Delta E}} \tag{5}$$

where ΔE is the effective barrier height (band offset minus confinement energy). For our system, $\Delta E \approx 200\,\text{meV}$ yields $b \approx 1$–$2\,\text{nm}$. In the **strong confinement regime**, where the confinement width $\ell_X \approx a_B$, the COM and relative motions become coupled, allowing for the electrostatic "squeezing" of the internal relative wavefunction.

4.4 Radiative and Non-Radiative Decay Dynamics

The measured total lifetime τ is determined by the sum of radiative (Γ_{rad}) and non-radiative (Γ_{nonrad}) decay rates:

$$\frac{1}{\tau} = \frac{1}{\tau_{\text{rad}}} + \frac{1}{\tau_{\text{nonrad}}} \tag{6}$$

Radiative Decay: According to Fermi's Golden Rule, the radiative rate scales with the square of the spatial overlap integral $\mathcal{O}_{eh}$ of the evanescent tails:

$$\tau_{\text{rad}}(d) = \tau_0 \exp\left(\frac{2d_{\text{e-h}}}{b}\right) \tag{7}$$

where $\tau_0 \approx 1\,\text{ps}$ is the intralayer contact lifetime.

Non-Radiative Decay: We estimate τ_{nonrad} by referring to 2D trions. While intralayer trions exhibit fast Auger-dominated lifetimes ($\sim$ 20–100 ps), the spatial separation in our 1D junction suppresses these channels, similar to interlayer trions in vertical stacks ($\sim$ 1–10 ns). The fact that our measured τ remains in the nanosecond regime and tracks perfectly with the spectral blue shift confirms that the dynamics are dominated by the gate-induced modulation of τ_{rad}.

4.5 Extraction of Dipole Length via Zero-Parameter Scaling

To extract the dynamic dipole length $d_{\text{e-h}}$ from the measured lifetime τ, we anchor the model to a reference state at charge neutrality ($V_{\text{BG}} = 0$, lifetime τ_1, dipole length d_1). By assuming the change in lifetime is primarily radiative:

$$\ln\left(\frac{\tau_1}{\tau_0}\right) = \frac{2d_1}{b}, \quad \ln\left(\frac{\tau}{\tau_0}\right) = \frac{2d_{\text{e-h}}}{b} \tag{8}$$

Taking the ratio eliminates the unknown tunneling length b, yielding the **zero-parameter relationship**:

$$d_{\text{e-h}} = d_1 \frac{\ln(\tau/\tau_0)}{\ln(\tau_1/\tau_0)} \tag{9}$$

This mapping allows us to convert the observed temporal dynamics into a structural measurement of the exciton's internal size. The quantitative agreement between the predicted energy shifts and the experimental data (Fig. 4 **c**) provides definitive evidence for the dipole-collapse mechanism.

5 Diffusion measurements

We present in this section the experimental conditions for measuring the anisotropic diffusion of the LI excitons.
The excitation laser for PL measurements is a diode laser from Thorlabs, centered at 730 nm. To observe diffusion, we focus it on the sample and look at the wide field image through a Flir camera, conjugated with the sample through two lenses, the objective one, and an infinite-conjugated lens in front of the camera. We filter out the laser light by introducing a short pass filter at 750 nm on the excitation path, and putting two long pass filters at 750nm and 800nm in front of the camera lens. In this configuration, the laser light is suppressed by an OD 18 above 750 nm, and OD 12 at 730 nm. The trions and neutral excitons signals coming from the bulk are also cut by the longpass at 800nm.

6 Theoretical Framework for 1D Exciton Diffusion

6.1 1D Diffusion-Recombination Equation

The spatiotemporal dynamics of the interfacial exciton density, $n(y, t)$, along the junction (y-axis) is described by the 1D diffusion-recombination equation:

$$\frac{\partial n(y,t)}{\partial t} = D_y \frac{\partial^2 n(y,t)}{\partial y^2} - \frac{n(y,t)}{\tau} + G(y,t) \tag{10}$$

where D_y is the 1D diffusion coefficient, τ is the total exciton lifetime, and $G(y, t)$ is the generation rate determined by the laser excitation profile. The transverse confinement ($\ell_x \approx 2.8\,\text{nm}$) is sufficiently strong that the motion is strictly restricted to the 1D coordinate y.

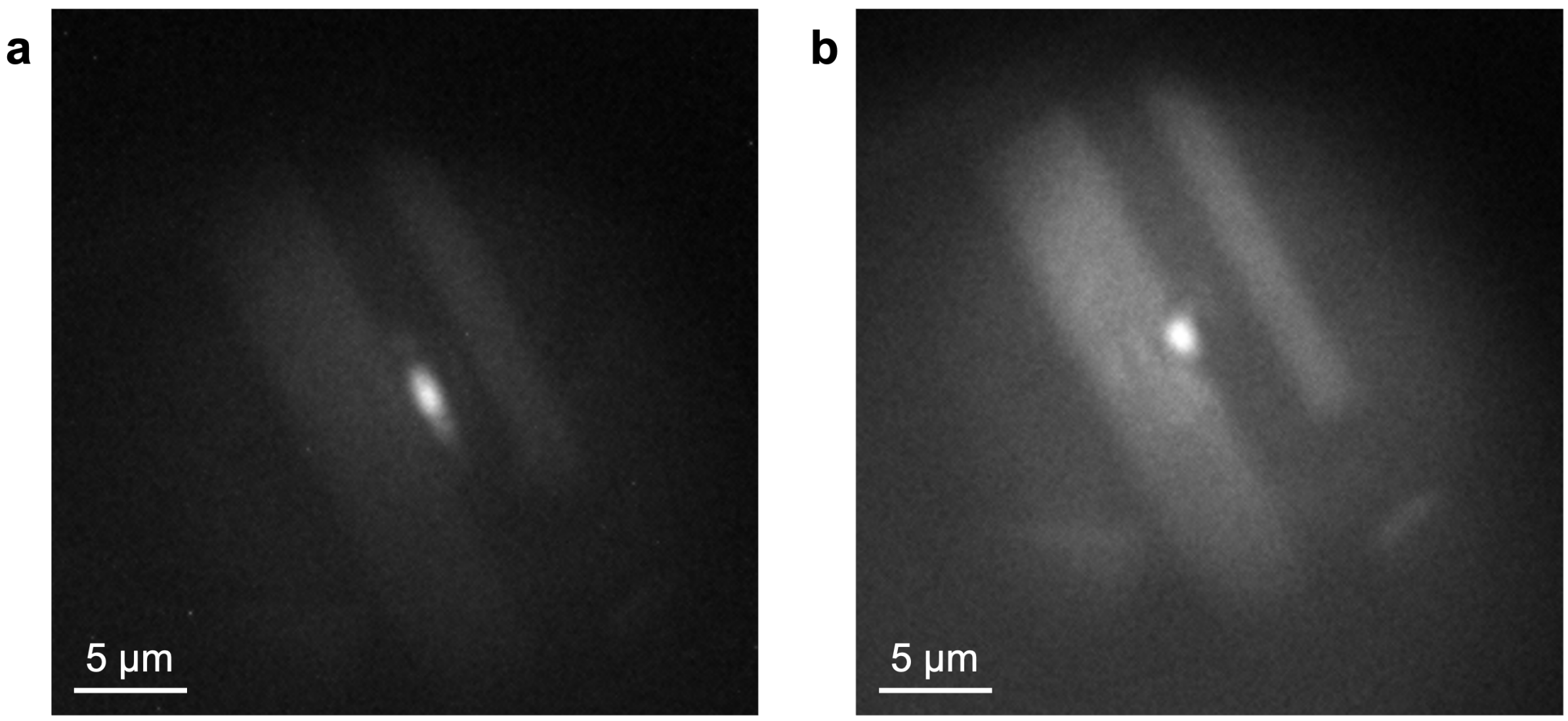

Figure 7: **Diffusion of LI excitons a** micrograph of the diffusion spot, as well as the gold split gates obtained by wide field imaging of the sample, with a long pass filter at 800nm and a long pass filter at 750 nm. The imaging light as an emission centered at 780nm. **b** Micrograph of the laser spot position for the diffusion experiment. It appears brighter and sitting on the left split gate due to the enhanced reflectivity of gold compared to hBN.

1. Diffusion Coefficient and Mobility Extraction

The longitudinal diffusion coefficient D_y is derived from the steady-state spatial profile of the interfacial exciton (X_{LI}) emission. The measured longitudinal width σ_y is a convolution of the intrinsic diffusion spread and the system point-spread function (PSF). The diffusion emission spot is fitted by two orthogonal gaussian curves $A\exp\left(\frac{-x^2}{2\sigma^2}\right)$, and both direction's radial standard deviation are extracted from the fit. The intrinsic diffusion length L_D is obtained via:

$$L_D = \sqrt{\sigma_y^2 - \sigma_{\mathrm{PSF}}^2} \tag{11}$$

Given the measured exciton lifetime τ, the diffusion coefficient in a steady-state regime is defined as:

$$D_y = \frac{L_D^2}{\tau} \tag{12}$$

Using $L_D \approx 0.7\,\mu$m and $\tau \approx 15\,$ns, we find $D_y \approx 0.4\,\mathrm{cm}^2/\mathrm{s}$. In the limit of low excitation density, the relationship between diffusivity and mobility μ_X is governed by the Einstein relation:

$$\mu_X = \frac{eD_y}{k_{\mathrm{B}}T} \tag{13}$$

At $T = 4\,$K, this yields an extracted mobility of $\mu_X \approx 1,200\,\mathrm{cm}^2/\mathrm{Vs}$.

6.2 Thermalization and Validity Assumptions

For the application of the Einstein relation, we assume the excitons reach thermal equilibrium with the lattice. The thermalization timescale (τ_{therm}) in TMDs is typically in the range of 10–100 ps, involving acoustic phonon scattering. Given that our measured lifetime $\tau \approx 15$ ns satisfies $\tau \gg \tau_{\text{therm}}$, the X_{LI} state is considered well-thermalized at the point of measurement, validating the use of the lattice temperature T in our mobility calculations.

7 Field-dependent and doping-dependent measurements

We justify here the different regimes of measurement that we have shown in the text. We prove that the sample was overall neutral for the dipole length measurement and that shifts were not doping induced.

Supplementary Information Fig. 8 and Supplementary Information Fig. 9 summarize the doping dependence of Device 1 as a function of the left and right split-gate voltages. Supplementary Information Fig. 8 presents a grid of PL intensity maps obtained by integrating the spectra over five excitonic windows: X_{LI}, X_{Mo}, X^{-}_{Mo}, X_{W} and X^{-}_{W}. The recurrence of high-intensity regions at the same gate-voltage sectors across all maps indicates that the device reaches equivalent doping states for the different excitonic species. The gate configuration employed for the split-gate Stark measurement lies within the charge-neutral sector identified in these maps.

Supplementary Information Fig. 9 presents spectral linecuts from the same datasets as Supplementary Information Fig. 8, with the right split-gate voltage fixed at the value used for the Stark measurements while the left split-gate is swept. The full PL spectra are retained for each voltage step: the central voltage bin used for the Stark analysis is clearly charge-neutral (neutral exciton dominates), whereas neighboring voltages are trion-dominated, indicating local doping on either side. The observation of the same charge-neutral window across all excitonic species demonstrates that our dipole-size extraction is not affected by unintended doping. This reproducible neutrality can be attributed to the device geometry: a narrow $MoSe_2$ channel (~ 50 nm) laterally enclosed by WSe_2, so that the local charge state of the sharp $MoSe_2$ interface is set by the surrounding WSe_2. Consequently, the device architecture provides stable control of the doping landscape and allows a clean separation of electric-field-induced Stark shifts from carrier-density-dependent spectral shifts. Moreover, the Stark shift produced by the split gates is small compared to the carrier-induced energy shifts and constitutes only a minor perturbation to the system.

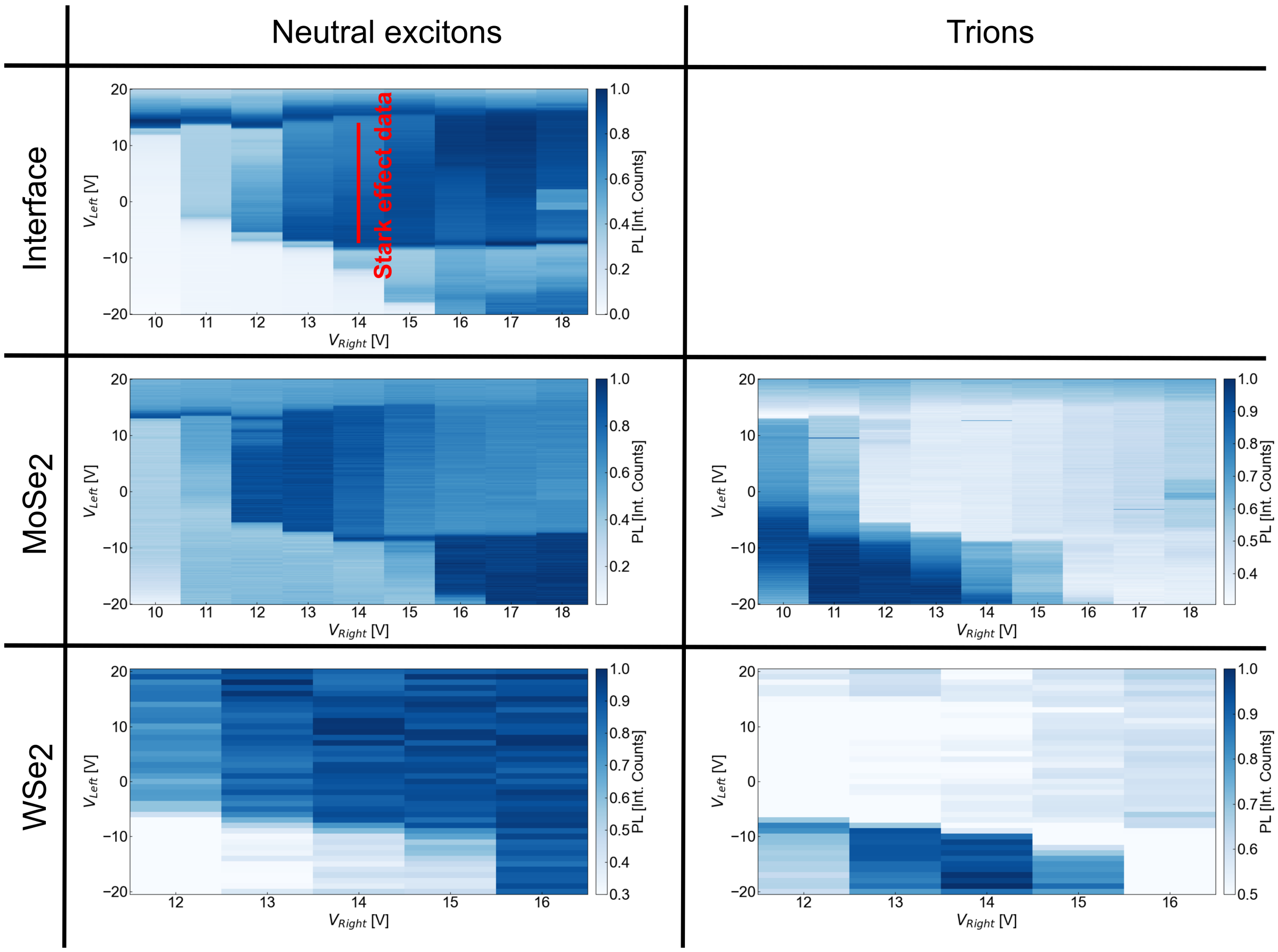

Figure 8: **Split-gate-dependent doping behavior of Device 1.** Grid of PL intensity maps as a function of both left and right split gate voltages, with each colormap representing PL spectra integrated over a specific energy range corresponding to different excitonic states. The axes of each plot correspond to the two independent split gate voltages, and the colormaps are organized from left to right and top to bottom according to the integration energy:

– X_{LI}: 1.54 ± 0.02 eV
– X_{Mo}: 1.64 ± 0.005 eV
– X_{Mo}^{-}: 1.61 ± 0.005 eV
– X_{W}: 1.72 ± 0.005 eV
– X_{W}^{-}: 1.69 ± 0.005 eV.

The recurrence of high-intensity regions at the same gate-voltage sectors across all maps indicates that the device reaches equivalent doping states for the different excitonic species. The gate configuration used for the split-gate Stark measurement lies within the charge-neutral region identified here.

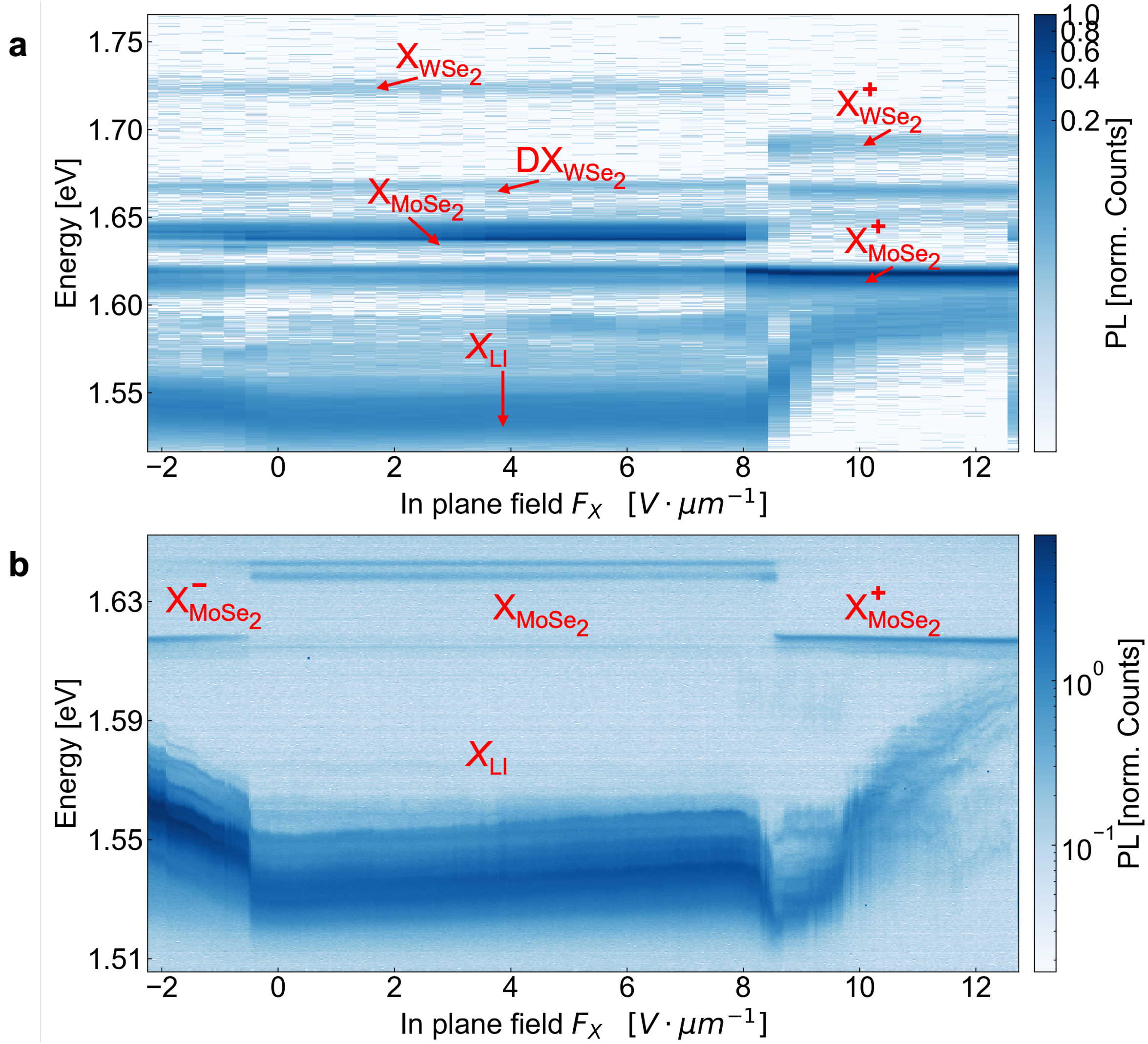

Figure 9: **Doping conditions of Device 1 for the Stark effect measurement**. **a, b** PL line-cuts from the same datasets as Supplementary Information Fig. 8 recorded at fixed right split-gate voltage $V_{\mathrm{Right}} = 14\,\mathrm{V}$ while sweeping the left split gate. PL spectra are shown for each voltage step and illustrate the doping state of the sample throughout the Stark effect measurement. The central voltage window used for the Stark analysis is clearly shown as charge-neutral by the dominance of the neutral $MoSe_2$ exciton—whereas adjacent voltage regions are trion-dominated, indicating local doping. These data confirm that the observed Stark effect arises solely from the applied electric field and is not influenced by doping variations.

8 Lifetime measurements

We present in this section additional results and considerations on the lifetime gate-dependence
For the lifetime measurements, long pass filters are used to filter out the exciton and trion emission from $\mathrm{MoSe_2}$, the back gate voltage is then scanned for a few values and the time trace is measured for the X_{LI} state only. The data is normalized and the background is subtracted, in order to perform a simple exponential fit. We emphasize the point that this measurement is done on all the center-of-mass quantized X_{LI} states at once, and that it represents an average of the possibly different lifetimes that emerge from them. For the zero parameter model that we use to relate the lifetime to the dipole (see section 4.3-5), we thus consider the average dipole length of the quantized ladder states to be 2nm at charge neutrality. From this reference point, we can infer the different average dipole lengths of the X_{LI} state for different charge densities and extract the hyper polarizability trend.

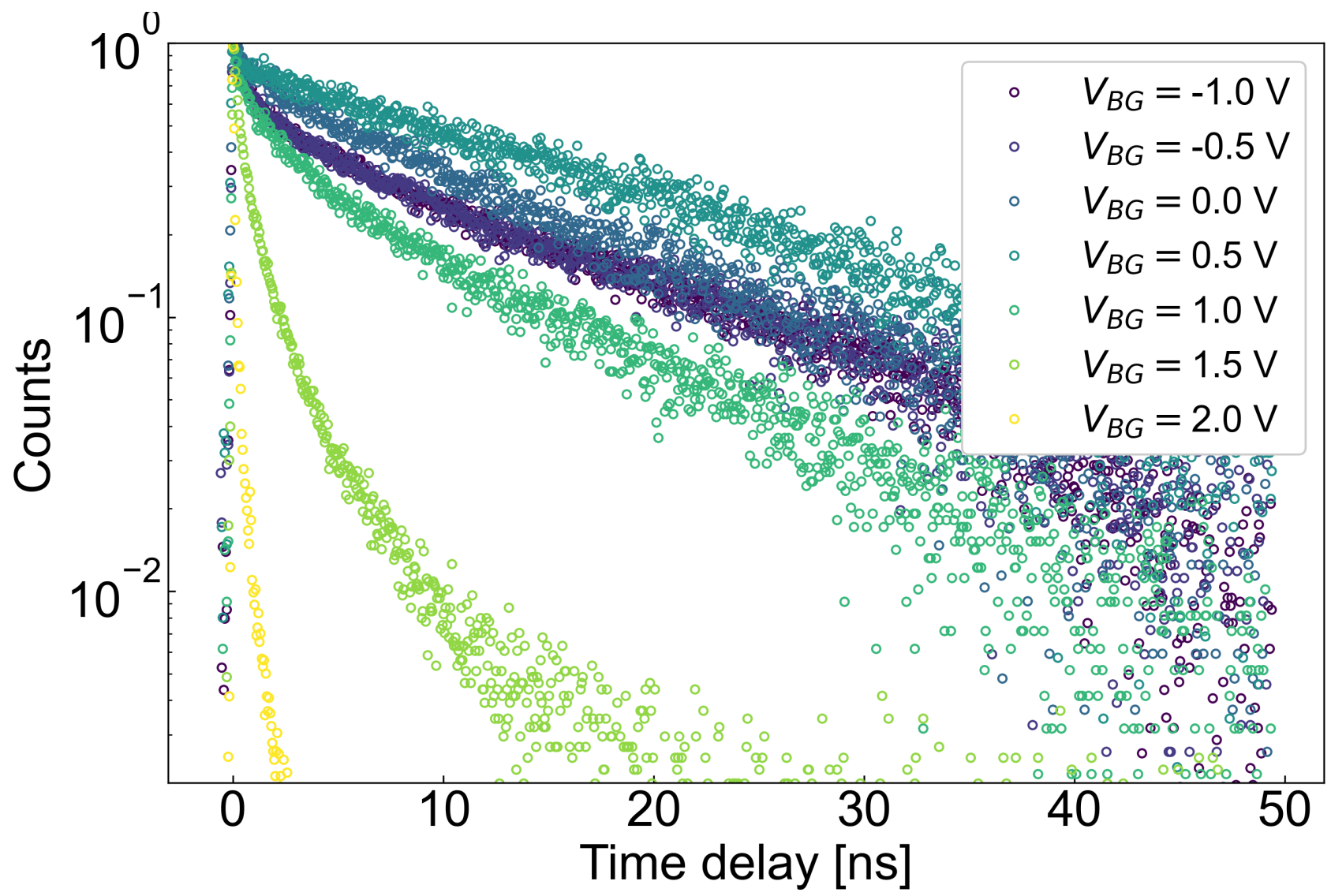

Figure 10: **Gate dependence of the lifetime of LI excitons** Each time trace is then fit by an exponential decay law $I = Ae^{\frac{-t}{\tau}}$, where τ is the lifetime of the exciton. The observed lifetime is the result of the averaging of all the quantum-confined states of dipole lengths varying from 1.6 to 2.3 nm. We use an average dipole length of 2nm corresponding to the lifetime of the neutral state in the model of figure 4.

9 Power measurements

We present in this section additional results and considerations on power saturation measurements

We present excitation power-dependent PL measurements for the direct 2D exciton X_{Mo} and the interfacial exciton X_{LI}, spanning more than two orders of magnitude in excitation power. While the 2D exciton exhibits the expected linear dependence on excitation power, the X_{LI} state shows a clear sub-linear power-law dependence (P^{α}) at higher powers, with a fitted exponent of α = 0.72. This suggests saturation effects possibly arising from a combination of tight COM confinement and repulsive interactions between permanent dipoles. Notably, this power-law scaling is in the intermediate regime between 2D and 0D, where the power dependence is slower than the linear scaling of 2D excitons but faster than the perfect saturation behavior typically seen in 0D quantum dots or defects. This further corroborates the 1D nature of the system. Further studies on nonlinear effects could uncover collective dipolar phenomena in this 1D system.

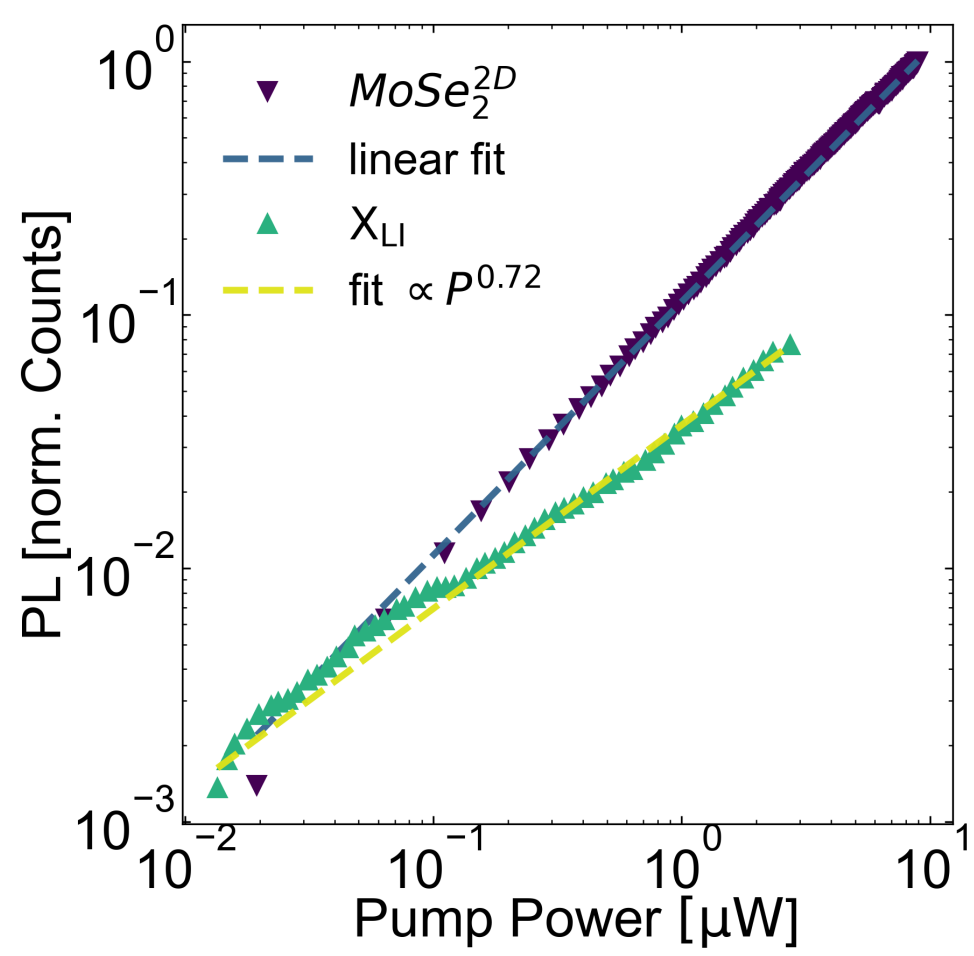

Figure 11: **Power dependence of the LI exciton state** Excitation power dependence of X_{LI} and X_{Mo} emission intensity: the direct exciton shows linear behavior, while the interface exciton follows a sublinear power law with an exponent of 0.72, indicating emission saturation due to confinement and dipolar interactions.

10 Electrostatic Simulation of LHS Device

We describe how the electrostatic simulations of the device are performed.
In this section we discuss the finite-element method (FEM) calculations of the device, which provide quantitative information of the in-plane fields and charge densities at a type-II TMDCs lateral interface. These computations are performed using the Electrostatics package in COMSOL. For all our simulations, we assume temperature T = 0 K.
Each TMDCs is modeled using the Thomas-Fermi approximation as a single sheet of charge with density,

$$\sigma_{\mathrm{XSe_2}}(x) = \sigma_{n,\mathrm{XSe_2}}(x) + \sigma_{p,\mathrm{XSe_2}}(x)$$

where X=Mo,W and $\sigma_{n,\mathrm{XSe_2}}(x)$ and $\sigma_{p,\mathrm{XSe_2}}(x)$ are electron and hole charge densities respectively, which are–in turn–given by,

$$\begin{aligned}\sigma_{n,\mathrm{XSe_2}}(x) &= -e\int_{E_{C,\mathrm{XSe_2}}(V(x))}^{E_F} D_{\mathrm{XSe_2}}\,dE,\; E_F > E_{C,\mathrm{XSe_2}}(V(x))\\ &= -e\,D_{\mathrm{XSe_2}}(E_F - E_{C,\mathrm{xSe_2}}),\end{aligned}$$

$$\begin{aligned}\sigma_{p,\mathrm{XSe_2}}(x) &= e\int_{E_F}^{E_{V,\mathrm{XSe_2}}(V(x))} D_{\mathrm{XSe_2}}\,dE,\; E_F < E_{V,\mathrm{XSe_2}}(V(x))\\ &= e\,D_{\mathrm{XSe_2}}(E_{V,\mathrm{xSe_2}} - E_F).\end{aligned}$$

Here $D_{\mathrm{XSe_2}} = g_S\,g_V\,m^*_{\mathrm{XSe_2}}/2\pi\hbar^2$ is the 2D density of states for electrons and holes in the TMD semiconductor, where $g_S = 1$ is the spin degeneracy and $g_V = 2$ is the valley degeneracy. $E_{C,\mathrm{XSe_2}}$ and $E_{V,\mathrm{XSe_2}}$ are the conduction and valence band edge energies, respectively, which depend on the local electrostatic potential $V(x)$ and on the type-II band alignment at the $MoSe_2$-WSe_2 interface. E_F is the Fermi level determined by the alignment of the contact work function with respect to the band edges.
The LHS sheet of charge is encapsulated by two 30-nm-thick hBN slabs and contacted by ohmic electrodes. The material parameters assumed for this calculation are as follows:

- WSe_2: bandgap $E_{b,\mathrm{WSe_2}} = 1.89\,\mathrm{eV}$, electron effective mass $m^*_{e,\mathrm{WSe_2}} = 0.4\,m_0$, hole effective mass $m^*_{h,\mathrm{WSe_2}} = 0.5\,m_0$, [4, 5], Fermi level offset relative to the valence band edge at zero potential $E_F - E_{V,\mathrm{WSe_2}}(V=0) = 0.80\,\mathrm{eV}$ [6]

- $MoSe_2$: bandgap $E_{b,\mathrm{MoSe_2}} = 1.87\,\mathrm{eV}$, electron effective mass $m^*_{e,\mathrm{MoSe_2}} = 0.8\,m_0$, hole effective mass $m^*_{h,\mathrm{MoSe_2}} = 0.6\,m_0$ [7], Fermi level offset relative to the valence band edge at zero potential $E_F - E_{V,\mathrm{MoSe_2}}(V=0) = 1.01\,\mathrm{eV}$ [6]

- h-BN: out-of-plane dielectric constant $\epsilon_\perp = 3.8$ and in-plane dielectric constant $\epsilon_\parallel = 6.9$ [8]

To avoid numerical instabilities from abrupt changes of material parameters at the $MoSe_2$–WSe_2 boundary, we introduce a transition region of 1 nm width in which the conduction and valence band edges and the electron and hole effective masses vary smoothly between the two materials. The numerical smoothness is ensured by using an hyperbolic tangent function to describe the parameters spatial variation.
Finally, the bottom gate and split gates are defined in the simulation as fixed-potential electrodes, and voltages are applied to obtain the spatial charge density and electric field distributions across the LHS. The simulation results in Fig. 4 of the main text are obtained by varying the bottom gate voltage V_{BG} to dope across the $MoSe_2$-WSe_2 interface. Separate simulations with only the split gates verify that the electric field at the junction is purely in-plane.

11 Data Analysis Procedure

We detail the PL data analysis procedure.
The PL intensity as a function of energy, $I(E)$, is modeled as the sum of seven Voigt profiles:

$$I(E) = \sum_{i=1}^{7} A_i \cdot V(E; E_{0,i}, \sigma_i, \gamma_i)$$

where the Voigt profile $V(E; E_{0,i}, \sigma_i, \gamma_i)$ is defined as the convolution of a Gaussian and a Lorentzian:

$$V(E; E_0, \sigma, \gamma) = \int_{-\infty}^{\infty} G(E'; E_0, \sigma) \cdot L(E - E'; E_0, \gamma)\, dE'$$

with

$$G(E; E_0, \sigma) = \frac{1}{\sigma\sqrt{2\pi}} \exp\left(-\frac{(E - E_0)^2}{2\sigma^2}\right)$$

$$L(E; E_0, \gamma) = \frac{\gamma}{\pi\left[(E - E_0)^2 + \gamma^2\right]}$$

Parameters:

- A_i: amplitude of the ith Voigt component
- $E_{0,i}$: center energy of the ith peak
- σ_i: Gaussian (standard deviation) component of the ith peak
- γ_i: Lorentzian (half-width at half-maximum) component of the ith peak

Supplementary Fig. 12 gives a visualization of the individual voigt profiles summing up to the spectral profile shown in Fig. 3 of the main text. The average Lorentzian linewidth extracted from these peaks is $\bar{\gamma} = 2\,\text{meV}$.

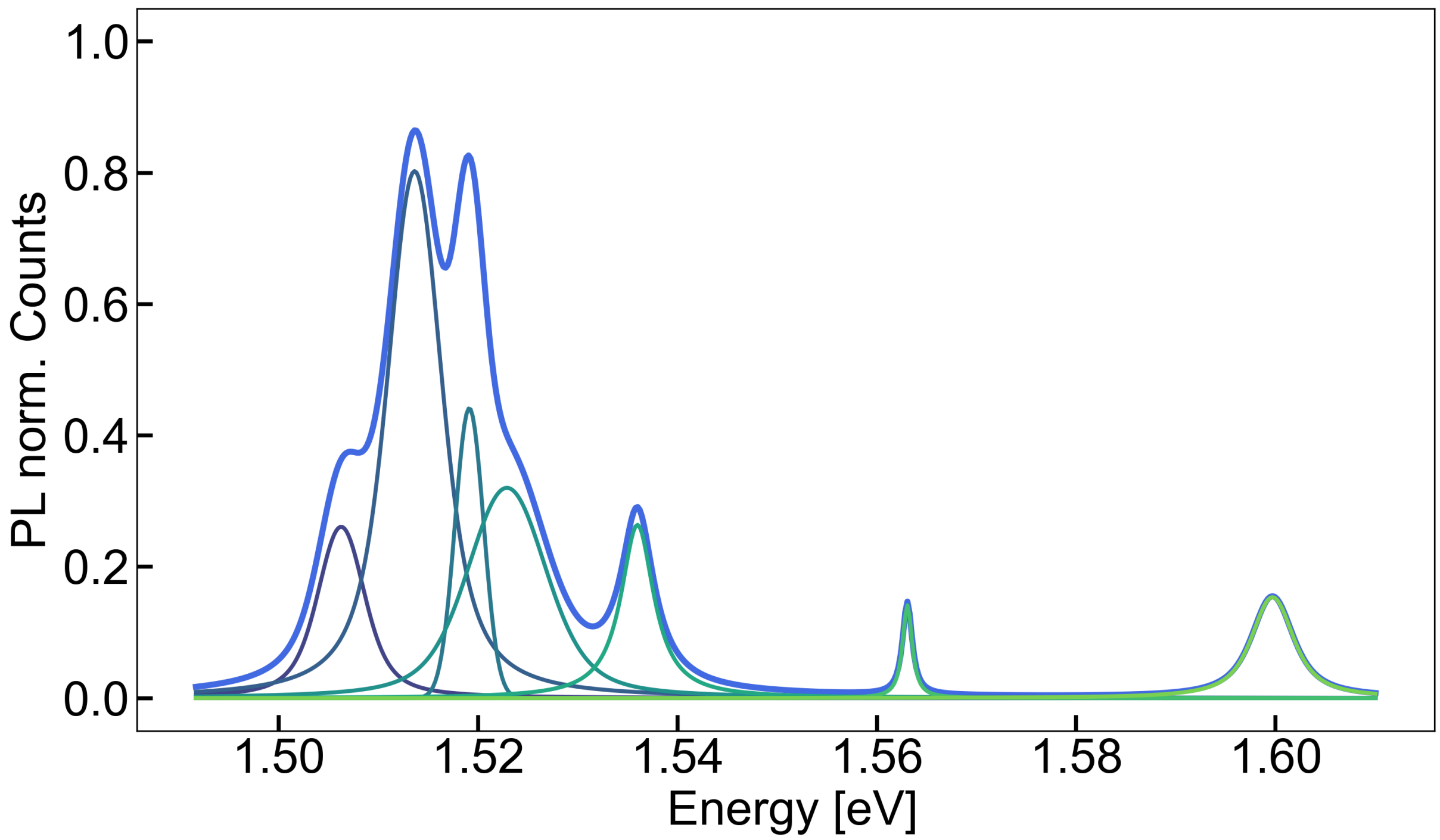

Figure 12: **Individual components of PL spectrum fit**. Seven individual Voigt profiles used to fit a PL spectrum are shown as thin lines, and their sum is plotted as the thick blue curve. The five lowest peaks are the one shown in the main text and the methods section.

12 Dipole Moment Uncertainty

We compute the LI exciton dipole moment uncertainty.
The dipole moment of the nth quantized state is extracted by fitting the split-gate-induced Stark shift data according to $E_n = E_n^0 + e\, d_{e\text{–}h}^{(n)}\, F_X$, where the field is given by $F_X = \frac{\Delta V}{d_{\text{SG}}}$, ΔV is the voltage difference between the split gates and $d_{\text{SG}} = 2.70\,\mu$m their separation. We assign $\delta d_{SG} = 0.13\,\mu$m uncertainty to the split-gate separation; uncertainties from the voltage source and the spectral fits are negligible in comparison. Consequently the dominant contribution to the dipole uncertainty is geometric and is the same for all quantized states. The dipole moment relative uncertainty is therefore:

$$\frac{\delta d_{e\text{–}h}^{(n)}}{d_{e\text{–}h}^{(n)}} = \frac{\delta d_{\text{SG}}}{d_{\text{SG}}} \simeq 5\%.$$

13 Distinguishing Interfacial Excitons from Defect-Bound States

We distinguish the interfacial exciton (X_{LI}) from localized defect states through five primary physical signatures:

1. **Spatial Delocalization and 1D Transport:** Unlike point-like defect states, which are spatially stationary, X_{LI} exhibits long-range 1D transport. The measured diffusion length ($L_D \approx 0.7\,\mu\mathrm{m}$) and high mobility ($\mu_X \approx 1,200\,\mathrm{cm}^2\mathrm{V}^{-1}\mathrm{s}^{-1}$) indicate a delocalized Bloch-like state moving along the crystallographic junction.

2. **Spatially Uniform Quantized Ladder:** Defects produce stochastic, irregularly spaced peaks that vary from site to site. In contrast, X_{LI} displays a highly ordered "ladder" of states with uniform spacing ($\Delta E \approx 7.2\,\mathrm{meV}$). This ladder is **reproduced at different spatial locations** along the entire interface, confirming the quantization is an intrinsic property of the global heterojunction.

3. **Synchronous Dipole Collapse:** The X_{LI} state exhibits a smooth, monotonic blue shift (Stark effect) up to $40\,\mathrm{meV}$, perfectly synchronized with a recursive lifetime collapse. This deterministic tuning is a unique consequence of the Type-II interface and cannot be replicated by the erratic spectral wandering characteristic of point defects.

4. **Power-Dependent Saturation:** Due to a finite density of trapping sites, defect luminescence typically saturates at low excitation powers ($P < 1\,\mu\mathrm{W}$). In contrast, the X_{LI} intensity shows a power-law dependence on power, showing that the state is intrinsic to the interfacial atomic chain rather than a sparse population of traps.

5. **Temporal Stability and Absence of Blinking:** Localized defect states in TMDs frequently exhibit "blinking" (intensity fluctuations) and spectral wandering on millisecond to second timescales due to local charge noise. In contrast, the X_{LI} emission is **temporally stable**, maintaining constant intensity and peak position over extended measurement windows. This stability confirms that the emission originates from a stable, crystallographically defined quasiparticle.

References

[1] Prasana K. Sahoo et al. "One-pot growth of two-dimensional lateral heterostructures via sequential edge-epitaxy". In: *Nature* 553.7686 (2018), pp. 63–67.

[2] J. Klein et al. "Site-selectively generated photon emitters in monolayer MoS2 via local helium ion irradiation". In: *Nature Communications* 10.1 (June 2019), p. 2755.

[3] Ajit Srivastava et al. "Optically active quantum dots in monolayer WSe2". In: *Nature Nanotechnology* 10.6 (June 2015), pp. 491–496.

[4] A. V. Stier et al. “Magnetooptics of Exciton Rydberg States in a Monolayer Semiconductor”. In: *Phys. Rev. Lett.* 120 (5 2018), p. 057405.

[5] Shao-Yu Chen et al. “Luminescent Emission of Excited Rydberg Excitons from Monolayer WSe2”. In: *Nano Letters* 19.4 (2019), pp. 2464–2471.

[6] Yuzheng Guo and John Robertson. “Band engineering in transition metal dichalcogenides: Stacked versus lateral heterostructures”. In: *Applied Physics Letters* 108.23 (2016), p. 233104.

[7] M. Goryca et al. “Revealing exciton masses and dielectric properties of monolayer semiconductors with high magnetic fields”. In: *Nature Communications* 10.1 (2019), p. 4172.

[8] Akash Laturia, Maarten L. Van de Put, and William G. Vandenberghe. “Dielectric properties of hexagonal boron nitride and transition metal dichalcogenides: from monolayer to bulk”. In: *npj 2D Materials and Applications* 2.1 (2018), p. 6.
